# Multilayer graphene shows intrinsic resistance peaks in the carrier density dependence

**DOI:** 10.1038/s41598-018-32214-7

**Published:** 2018-09-18

**Authors:** Taiki Hirahara, Ryoya Ebisuoka, Takushi Oka, Tomoaki Nakasuga, Shingo Tajima, Kenji Watanabe, Takashi Taniguchi, Ryuta Yagi

**Affiliations:** 1 Graduate School of Advanced Sciences of Matter, Hiroshima University, 1-3-1, Kagamiyama Higashi-Hiroshima, Hiroshima, 739-8530 Japan; 2 National Institute for Materials Science (NIMS), 1-1, Namiki, Tsukuba, Ibaraki 305-0044 Japan

## Abstract

Since the advent of graphene, a variety of studies have been performed to elucidate its fundamental physics, or to explore its practical applications. Gate-tunable resistance is one of the most important properties of graphene and has been studied in 1–3 layer graphene in a number of efforts to control the band gap to obtain a large on-off ratio. On the other hand, the transport property of multilayer graphene with more than three layers is less well understood. Here we show a new aspect of multilayer graphene. We found that four-layer graphene shows intrinsic peak structures in the gate voltage dependence of its resistance at zero magnetic field. Measurement of quantum oscillations in magnetic field confirmed that the peaks originate from the specific band structure of graphene and appear at the carrier density for the bottoms of conduction bands and valence bands. The intrinsic peak structures should generally be observed in AB-stacked multilayer graphene. The present results would be significant for understanding the physics of graphene and making graphene FET devices.

## Introduction

**Figure 1:**
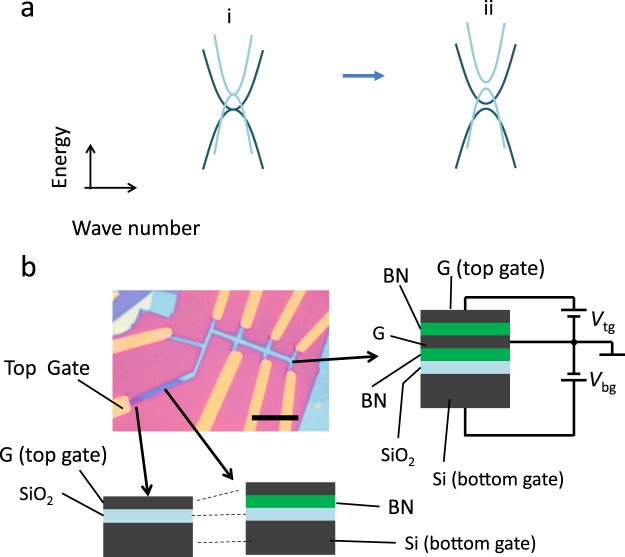
Band structure of tetralayer graphene and sample structure. (**a**) Simplified band structure in tetralayer graphene. i Tetralayer graphene consists of a set of light-mass and heavy-mass bilayer-like bands, which are offset in energy. ii Perpendicular electric field opens a band gap at the bottoms of the conduction and the valence bands. (**b**) Optical micrograph of encapsulated tetralayer graphene sample that has a top gate (Top left). The scale bar is 10 μm. Schematic structure of an encapsulated graphene stack is shown in the right panel. G is graphene, BN is *h*-BN, Si is Si substrate and SiO_2_ is SiO_2_ covering on the Si substrate. Tetralayer graphene was encapsulated with thin *h*-BN flakes, and it was formed on a SiO_2_/Si substrate. Several layer graphene, which served as a top gate, was deposited onto the top of the encapsulated graphene. Samples were ion-etched into Hall bar shape. Electric contact to the tetralayer graphene was formed by using the technique in ref.^15^. The lead that is indicated by “Top Gate” in the figure is connected to only the top gate graphene by using the structures illustrated by the two figures at the bottom.

Graphene is a material with excellent physical properties—its electrical mobility, for example, much higher than that of organic semiconductors^1,2^—and is a candidate base material for next-generation devices. Controllability of resistance by using gate voltage is one of most significant properties of graphene for practical application in FET devices. It is known that transport property of graphene is highly influenced by the surface environment (*e*.*g*., ionized impurity, contamination or ripples, *etc*.^3,4^). Gate-voltage dependence of the resistance of such dirty graphene samples often shows multiple-peak structure, which possibly originate from local potential energy differences due to the surface contamination. Placing graphene on high-quality *h*-BN flakes or using encapsulation techniques drastically improve the electron mobility of graphene samples and enables one to measure intrinsic features of the transport property of graphene^5–7^. In high-mobility graphene, a large resistance peak appears at the charge neutrality point and resistance decreases monotonically with increasing carrier density^5–7^. This would be a natural property of graphene with a single band, such as mono- and bilayer graphene. Although AB-stacked trilayer graphene has a bilayer-like band and a monolayer-like band, the gate voltage dependence of its resistance would be similar to that of the single-band system because the density of states of the bilayer-like band is much larger than that of the monolayer-like band and is dominant^7^. On the other hand, AB-stacked multilayer graphene with larger number of layers does not necessarily show the above mentioned property because it has more than two bilayer-like bands. In this work, we studied AB-stacked tetralayer graphene, which is the simplest case of multilayer graphene with more than three layers. The band structure of AB-stacked tetralayer graphene is shown schematically in panel i of Fig. 1a. The energy of the bottom of the light-mass bilayer-like band is higher than that of the heavy-mass bilayer-like band, and the density of states of the light-mass bilayer-like band is not negligible with respect to that of the heavy-mass bilayer-like band. This paper will show that this multi-bilayer-like band leads to the phenomenon that gate-voltage dependence of resistance generally shows intrinsic peak structures.

## Results

### Experimental

We employed a technique to modify electronic band structure by using perpendicular electric field. The method was originally proposed to create an energy gap in the graphene system. Monolayer graphene, where electrons behave like massless Dirac fermions with linear dispersion relation^8–10^, does not have a band gap. Bilayer graphene, where electron bands have massive dispersion relations^10–12^, does not have a band gap either. However, it was predicted that a perpendicular electric field forms an energy gap between the bottoms of conduction and valence bands^13,14^. The response of AB-stacked graphene’s electronic structure to perpendicular electric fields shows significant layer-number dependence. AB-stacked trilayer graphene, for example, has a bilayer-like band and monolayer-like band. Unlike bilayer graphene, it does not exhibit insulating behavior induced by applying a perpendicular electric field. Its electric resistance instead decreases^13–15^. Electronic band structure in AB-stacked tetralayer graphene in the presence of a perpendicular electric field is schematically described in panel ii in Fig. 1a. An electric field opens an energy gap at the bottoms of each of the bilayer-like bands.

In order to detect electronic structure by using a transport experiment, the quality of the graphene should be sufficiently high. Moreover, top and bottom electrodes are required to apply a perpendicular electric field. This requirement was attained by using the AB-stacked tetralayer graphene device shown in Fig. 1b. AB-stacked tetralayer graphene was encapsulated with *h*-BN^16^, and onto the top of the stack was transferred a rather thick graphene flake serving as a top gate electrode. This stack of films was formed on a SiO_2_/Si substrate, where Si is conducting at low temperatures and served as a bottom gate. Encapsulation of graphene by high-quality *h*-BN flakes drastically increases the electric mobility of graphene. The high-quality and atomically flat surface of the *h*-BN flakes reduces scattering due to ionized impurity and surface roughness^6,16,17^. Mobility of the graphene which was calculated with *μ* = 1/*nep*, was more than 40,000 cm^2^/VS at the high carrier density regime.

### Gate voltage dependence of resistivity at zero magnetic field

**Figure 2:**
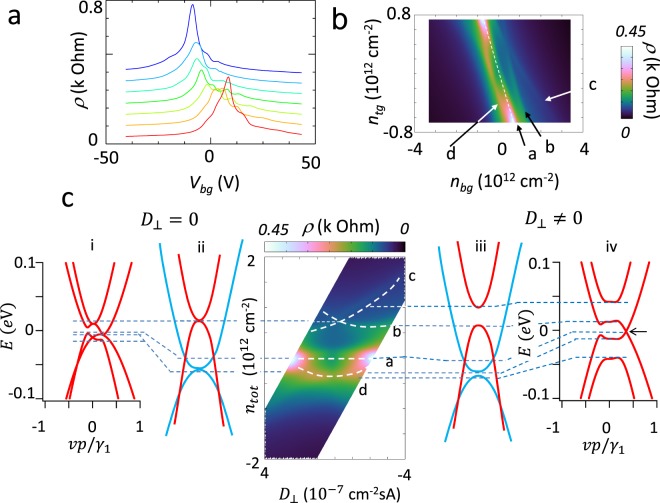
Gate voltage dependence of resistance at zero magnetic field. (**a**) Bottom gate voltage *V*_*bg*_ dependence of resistivity *ρ* for different values of top gate voltages *V*_*tg*_. From top to bottom, *V*_*tg*_ was varied from 7.5 V to −7.5 V in 2.5 V steps. *T* = 4.2 K. (**b**) Map of *ρ* as functions of carrier densities *n*_*bg*_ and *n*_*tg*_, which are tuned by the bottom gate and the top gate voltages and were converted from *V*_tg_ and *V*_*bg*_. Here, *T* = 4.2 K. *B* = 0 T. Arrows (**a**–**d**) show resistance ridge structure. The white dashed line is the trace of the charge neutrality point. (**c**) Replot of panel (**b**) against perpendicular electric flux density *D*_⊥_ and carrier density *n*_*tot*_. White broken lines (**a**–**d**) show positions of resistance ridges.

Figure 2a shows bottom gate voltage *V*_*bg*_ dependence of resistivity for different top gate voltages *V*_*tg*_ measured at *T* = 4.2 K. In the trace with *V*_*tg*_ = 0V, a slightly complicated peak structures are discernible near the charge neutrality point. The peak structure showed significant variation as top gate voltages was varied. The most prominent variation is enhancement of the peak, which grows with increasing |*V*_*tg*_| values and appeared roughly symmetric with respect to *V*_*tg*_ = 0*V*. The enhancement of the resistance by top gate voltage would be qualitatively understood by formation of a band gap by perpendicular electric fields, as in bilayer graphene^14^. However, the largest peak of *V*_*bg*_-dependence of resistivity at *V*_*tg*_ = 0*V* reached to only approximately 250 ohms, and this value was smaller than that in the preceding study in AB-stacked tetralayer graphene that reported insulating behavior^18^. This indicates that energy gap in tetralayer graphene encapsulated with *h*-BN would be considerably small (see the Supplementary Information).

Beside the large peak structure there were small peaks as shown in Fig. 2a. These peaks appeared even at *V*_*tg*_ = 0V. As shown in the following discussion, the presence of these peaks is an intrinsic property of AB-stacked tetralayer graphene. In the case of dirty graphene, multiple peaks might be observed because inhomogeneity in the sample or surface contaminations can create locally different electrostatic potentials. However, our samples were encapsulated using a dry process, which ensures clean surfaces. The peaks are reproducibly observed in other samples with the same number of layers. Samples with larger numbers of layers also show different peak structures (see Supplementary Information).

In order to study the peak structure further, we measured resistivity as a function of the top and the bottom gate voltages (Fig. 2b). Gate voltages were converted into carrier densities *n*_*tg*_ and *n*_*bg*_, which are induced by top and bottom gate voltage, and were calculated by using calibrated specific capacitance. It is clear that the observed peak in Fig. 2a showed significant dependence on *n*_*tg*_ and *n*_*bg*_, and appeared as resistance ridges. By applying both top and bottom gate voltage, total charge density −*en*_*tot*_ induced in graphene can be expressed by $-e{n}_{tot}=-\,e({n}_{tg}+{n}_{bg})=\,-\,({C}_{tg}{V}_{tg}+\,{C}_{bg}\,{V}_{bg})$ where *C*_*tg*_ and *C*_*bg*_ are top and bottom gate capacitances, respectively. The white broken line a in the figure that passes through *n*_*bg*_ = 0 and *n*_*tg*_ = 0 is a resistance ridge that connects two large peaks appearing at *n*_*tg*_ = ±0.8 × 10^12^. On this line, graphene is in a condition of charge neutrality and total carrier density estimated from Hall resistance vanishes. Here, carriers induced by the top gate voltage and bottom gate voltage are compensated, *i*.*e*., *C*_*tg*_*V*_*tg*_ + *C*_*bg*_*V*_*bg*_ = 0. The ratio of capacitances, *C*_*bg*_/*C*_*tg*_ was calculated to be 0.078. Using calibrated specific bottom gate capacitance, *C*_*bg*_ = 108 aF/μm^2^, *C*_*tg*_ was calculated to be *C*_*tg*_ = 1385 aF/μm^2^. On the other hand, *D*_⊥_ = −*e*(*n*_*bg*_ − *n*_*tg*_) = *C*_*tg*_*V*_*tg*_ − *C*_*bg*_*V*_*bg*_ is the difference in charge density induced by top and bottom gate voltages and is proportional to the electric flux density in graphene. *D*_⊥_ is proportional to the electric field perpendicular to the surface of graphene. At the charge neutrality point, ridge a appears. In addition, other resistance ridges b, c, and d showed different dependence on gate voltages. In particular ridges b and c are not parallel to the line of charge neutrality point (ridge a), and crossed at approximately ${n}_{bg}=\,{n}_{tg}\approx 0.4\times {10}^{12}\,{{\rm{cm}}}^{-2}$. The ridge d also show a weak dependence as is seen by connecting two largest resistance peaks appearing at *n*_*tg*_ = ±0.8 × 10^12^ cm^−2^.

In order to inspect the nature of the ridges, we replot it as a function of *n*_tot_ and electric flux density *D*_⊥_ as shown in Fig. 2c. The figure has a few remarkable features. First, ridge structures appear symmetrically about *D*_⊥_ = 0, from which one could infer that the ridge structures originated from the intrinsic property of graphene. In particular, ridges b and c overlap at *D*_⊥_ = 0, forming a single peak. These ridges possibly originated from bottoms of a conduction band and a valence band of light-mass bilayer-like band. At *D*_⊥_ = 0, the conduction band and valence band of the light-mass bilayer-like band contact at their bottoms (see left panel ii in Fig. 2c). The structure of the crossing is reminiscent of the energy gap in the bilayer-like band formed by applying a perpendicular electric field as shown in panel iii in Fig. 2c and is possibly related to the light-mass bilayer-like band. Secondly, ridge d seems to connect large peaks appearing at *D*_⊥_≈ ±2× 10^−7^ cm^−2^ sA and merge into ridge a. The growing resistivity with increasing |*D*_⊥_| at *n*_*tot*_ = 0 would result from decreasing semi-metallic carriers. Density of states for electron-like band and hole-like band tends to decrease. Possibly a small energy gap is formed (see the Supplementary Information).

### Landau level spectroscopy

**Figure 3:**
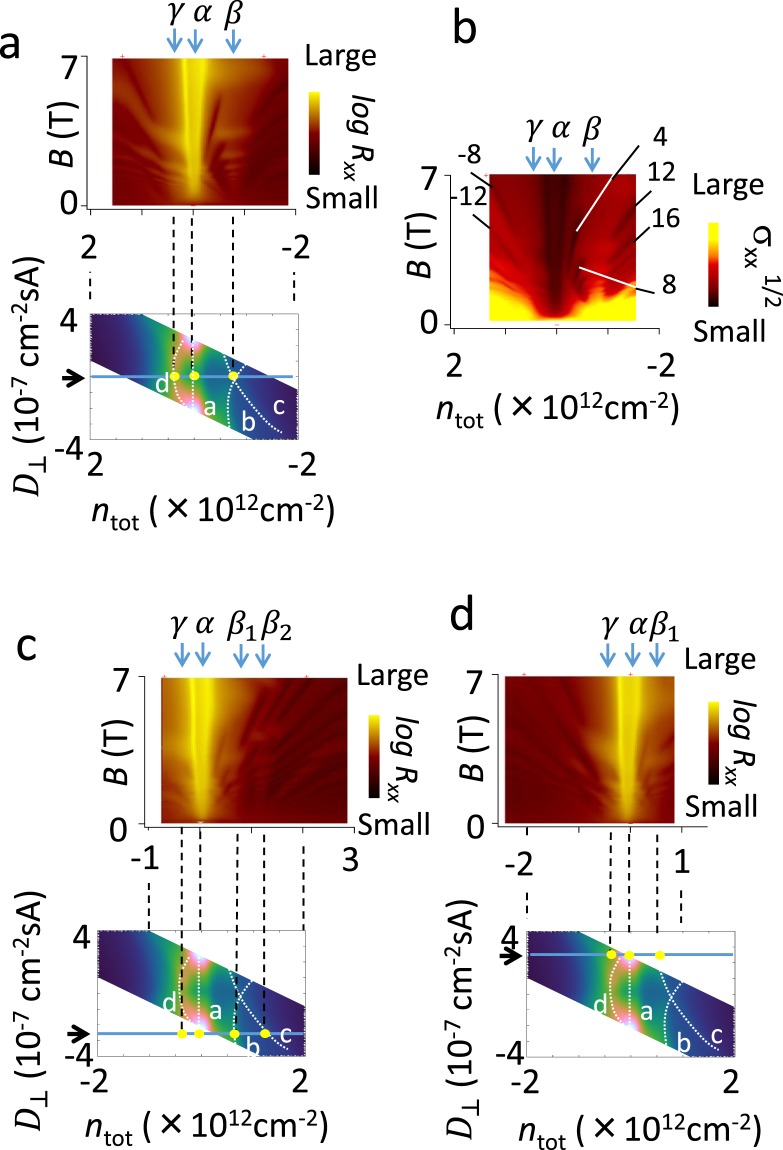
Landau fan diagram of tetralayer graphene. (**a**) (Top) Map of longitudinal resistivity *R*_*xx*_ at zero perpendicular electric flux density, *D*_⊥_ = 0. *T* = 4.2 K. *β* and *γ* indicate position of zero-mode Landau levels of light-mass bilayer-like band and heavy-mass bilayer-like band. α indicates position of energy gap which appears at the charge neutrality point. (Bottom) Resistance ridge structure at zero magnetic field (the same as Fig. 2c) Filled yellow circles indicate positions of the zero-mode Landau levels and charge neutrality point. (**b**) Map of *σ*_*xx*_ at *D*_⊥_ = 0. Filling factors for some gaps are shown. (**c**) Similar plot as panel a for *D*_⊥_ = −2.7 × 10^−7^ cm^−2^ sA. *β*_1_ and *β*_2_ indicate split zero-mode Landau levels of light-mass bilayer-like band. *T* = 4.2 K. (**d**) Similar plot as panel c for *D*_⊥_ = +2.7 × 10^−7^ cm^−2^ sA. *T* = 4.2 K.

Energy band structure can be directly probed by quantum oscillation measurement in magnetic fields at *T* = 4.2 K. The structure of Landau levels revealed the nature of the resistance ridges at zero magnetic field. Figure 3a displays a map of *R*_*xx*_ as a function of *n*_*tot*_ and *B*. Here *V*_*tg*_ and *V*_*bg*_ were varied so that the condition *D*_⊥_ = 0 was satisfied. Figure 3b shows a color map of longitudinal conductivity ${\sigma }_{xx}\,$which was calculated by using a formula, ${\sigma }_{xx}={\rho }_{xx}/({\rho }_{xx}^{2}+{\rho }_{xy}^{2})$. Before discussing the effect of perpendicular electric fields, here, we briefly explain the fan diagram. Complicated stripes are due to Shubnikov-de Haas oscillation which originated from Landau quantization of two-dimensional electrons. Each stripe is a Landau level of a specific band, and has a specific index. Landau levels of AB-stacked tetralayer graphene basically consist of two sets of Landau levels for bilayer-like bands: one for the light-mass bilayer-like band and the other for the heavy-mass bilayer-like band. Mixing and crossing of these Landau levels make the structure of the fan diagram complicated^18,19^. Next, we focus on zero-mode Landau levels that appear near the bottoms of bands, and energy gap appearing in the vicinity of charge neutrality points. Arrows *β* and *γ*, which grow vertically with increasing magnetic field, are zero-mode Landau levels of the light-mass and the heavy-mass bilayer-like band. These zero-mode Landau levels generally appear at the bottoms of bands in the Dirac fermions^9^. Analysis of filling factor *ν* of energy gaps associated with the Landau level crossing indicated that the zero-mode Landau levels have a degeneracy of eight, which is a hallmark of bilayer-like bands.

Now we discuss the relationship between the fan diagram and the ridge structure at zero magnetic field. Ridge structures appear approximately the same values of *n*_*tot*_ with those of zero-mode Landau levels. Because zero-mode Landau level is formed near the bottoms of the bands, *n*_*tot*_ at which the zero-mode Landau level appears would be a measure of *n*_*tot*_ for the bottoms of the bands at zero magnetic field. For *D*_⊥_ = 0, the zero-mode Landau level *β* of light mass bilayer-like band appeared at approximately the same *n*_*tot*_ as the crossing point of the ridges b and c in the lower panel in Fig. 3a (the same as Fig. 2c). On the other hand, zero-mode Landau level *γ* for heavy mass bilayer-like band appeared on ridge d in the vicinity of *D*_⊥_ = 0.

A similar correspondence can be found in the case of non-vanishing vertical electric field. Figure 3c,d show results for *D*_⊥_ = -2.7 × 10^−7^ and +2.7 × 10^−7^ cm^−2^ sA, respectively. Values of *D*_⊥_ are indicated by arrows in lower panels. It is clear that zero-mode Landau level *β* in Fig. 3a has split into two zero-modes *β*_1_ and *β*_2_ with four-fold degeneracy. (*β*_2_ also showed small splitting into two levels with two-fold degeneracy at high magnetic fields). The zero-mode Landau levels *β*_1_ and *β*_2_, appeared at approximately the same *n*_*tot*_ of that for ridges b and c in zero magnetic field (yellow points). As for the heavy-mass bilayer-like band, splitting is not clearly observed in the fan diagram. However ridge structures similar to a and b are actually observed for heavy mass bilayer-like band in measurement of wider area using a different sample (see the Supplementary Information).

Ridge a, on the other hand, is not relevant to zero-mode Landau levels. As seen in Fig. 3a,b, when at least *B* > 0.5 T, there was an energy gap at around *n*_tot_ = 0 (indicated with arrow α). This indicates that carrier density is considerably small in zero magnetic field. The ridge d in Fig. 3 is influenced by growing resistivity of ridge a for |*D*_⊥_| > ± 0.8 × 10^−7^ cm−2 sA.

### Behavior of zero-mode Landau levels with respect to *D*_⊥_ at a fixed magnetic field

**Figure 4:**
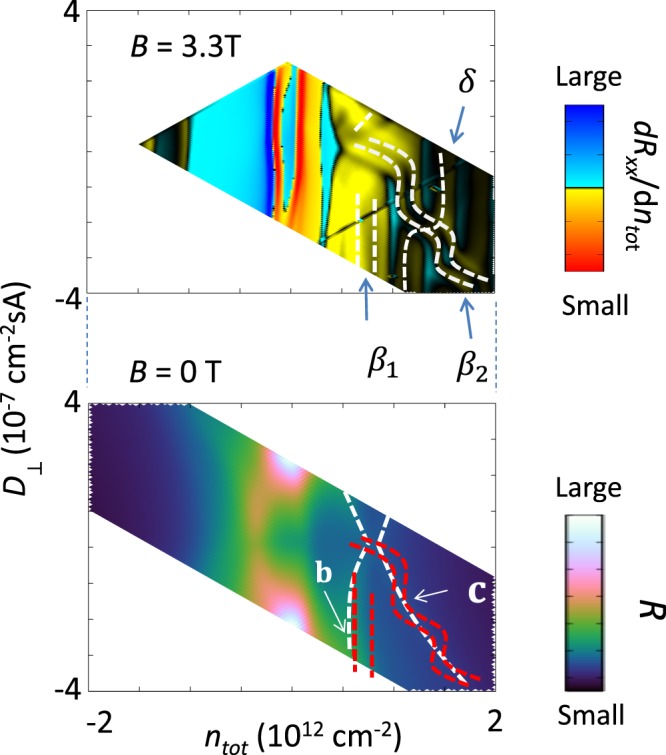
Top and bottom gate voltage dependences at *B* = 3.3 T and *B* = 0 T. (Top) A map of derivative of longitudinal resistivity *R*_*xx*_ with respect to total carrier density $\delta$ as a function of *n*_*tot*_ and *D*_⊥_ *B* = 3.3 T and *T* = 4.2 K. Dashed lines show positions of center of some Landau levels.* β*_1_ and *β*_2_ denote zero-mode Landau levels of light-mass bilayer-like band. *δ* is a Landau level with a higher index. *δ* and *β*_2_ cross at ${n}_{tot}\approx \,1.3\,\times \,{10}^{12}$ ${{\rm{cm}}}^{-2}$ and ${D}_{\perp }=-\,2\times {10}^{-7}$ ${{\rm{cm}}}^{-2}{\rm{sA}}$. (Bottom) Similar plot of the resistivity at *B* = 0 T and *T* = 4.2 K (same as Fig. 2c). Dashed red lines indicate position of zero-mode Landau levels *β*_1_ and *β*_2_.

The origin of the ridge structure was further checked by detailed measurements of Landau levels as a function of *n*_*tot*_ and *D*_⊥_. Upper panel in Fig. 4 displays a top and bottom gate voltage dependence of longitudinal resistivity measured in a magnetic field of approximately *B = *3.3 T. The derivative of longitudinal resistance with respect to total carrier density *n*_*tot*_ (*dR*_*xx*_/*dn*_*tot*_) is shown to enhance visibility. The step-like structure indicated by *β*_2_ corresponds to zero-mode Landau level *β*_2_ in Fig. 3c. Two dashed lines of the step shape indicate that Landau level *β*_2_ split. Zero-mode Landau level *β*_1_ also split into two levels. By comparing with lower panel in Fig. 4, which is results for zero magnetic field, it is clear that zero-mode Landau Levels *β*_1_ and *β*_2_ follow the ridge structure b and c. This clearly indicates that the ridges b and c originate from bottoms of split light-mass bilayer-like band. The step-like feature originates from crossing of zero-mode with other Landau levels with higher indices. For example, a step appearing at *D*_⊥_ ≈ −2 × 10^−7^ cm^−2^ sA and *n*_*tot*_ ≈ 1.3 × 10^12^ cm^−2^ is a crossing with the Landau level labeled as δ. On the other hand, the zero-mode Landau level of the heavy-mass bilayer-like band, is not clearly seen in Fig. 4. This is because at the measured magnetic field, the zero-mode Landau level crosses the Landau levels that appear between gaps with filling factors −12 and −8 at *n*_*tot*_ = −0.4 × 10^12^ cm^−2^.

## Discussion

### Origin of resistance ridge structure in zero magnetic field

As seen in the above, the ridges in the carrier density dependence of resistance (or resistance peaks in *n*_*tot*_ dependences at fixed values of *D*_⊥_) at zero magnetic field reflected electronic band structure. Ridges, which appeared not at the charge neutrality point, are closely related to the bottoms of conduction and valence bands. Conductivity of the samples is contributed by the light-mass bilayer-like band and the heavy-mass bilayer-like band, which are naturally expected to have different mobilities. Therefore, in the frame of two-carrier model conductivity σ is expressed by

1 $${\rm{\sigma }}=|{n}_{{\rm{L}}}|e{\mu }_{L}+|{n}_{H}|e{\mu }_{H}$$

Here *n*_*L*_ and *n*_*H*_ are carrier densities of the light-mass bilayer-like band and heavy-mass bilayer-like band, and *μ*_*L*_ and *μ*_*H*_ are their mobilities. Total carrier density is sum of *n*_*L*_ and *n*_*H*_, *i*.*e*., *n*_*tot*_ = *n*_*L*_ + *n*_*H*_. The values of the mobility may be different because the band masses of the light-mass bilayer-like band and heavy-mass bilayer-like band should be different. In order to discuss resistance peak structure (or conductance dip structure), one needs to assume *n*_*tot*_ dependence of *n*_*L*_ or *n*_*H*_. If we assume a semi-metallic band structure consisting of parabolic bands and assume that *μ*_*L*_ and *μ*_*H*_ are constant values, *n*_*tot*_ dependence of the conductance is simply given by a polygonal line whose vertices are located at *n*_*tot*_ for the bottoms of the bands. This results in discontinuous *dσ*/*dn*_*tot*_ at the vertices and generally results in a single dip structure in the conductivity (or a single peak in the resistance). To explain resistance peaks at the bottoms of the bands, the mobility for each band would presumably be dependent on the carrier density. Mobility calculated by using *μ* = σ/*en*_tot_ generally varies with the carrier density. This phenomenon is widely seen in monolayer and bilayer graphene and results from nature of ionized impurity scattering and screening^20^. Moreover, mobilities would vary at the bottoms of the bands because the band mass would vary near the bottoms of the band owing to mixing of light-mass and heavy-mass bilayer-like bands which is shown in a band calculation considering full Slonczweski-Weiss-McClure (SWMcC) parameters^21^ (see the Supplementary Information).

On the other hand, ridge structure, which was observed at the vicinity of charge neutrality point, would require a bit different explanation. The observed ridge at the charge neutrality point would be related to the report from ref.^18^ showing insulating behavior at the charge neutrality point. A small energy gap is possibly formed because of, for example, the staggered potential due to many body interaction of electrons^18,22,23^. In this paper we tentatively attributed the enhancement of resistance to possible formation of energy gap (see the Supplementary Information). Electronic states near the charge neutrality point in tetralayer graphene is expected to be complicated as compared to graphene with fewer number of layers, and would require further investigation.

### Spatial symmetry and energy gap

In case of even-layer graphene, spatial inversion symmetry and time reversal symmetry impose strict conditions on the energy state. A conduction band and a valence bands are degenerated at *k* = 0. Therefore, bottoms of conduction and valence bands contact at *k* = 0, and an energy gap does not open^23^. However if spatial inversion symmetry is broken by the potential due to a perpendicular electric field, an energy gap is allowed to open. This fact could be clearly observed by the crossing of ridges b and c at *D*_⊥_ = 0. Simple band calculation indicates that gap magnitude generally differs between light-mass and heavy-mass bilayer-like bands, as was observed in the experiment (see Supplementary Information).

### Valley splitting

The broken spatial symmetry caused by the perpendicular electric field is also relevant to the valley splitting of the zero-mode Landau levels. At zero magnetic field, spatial inversion symmetry results in degenerate quantum states for K and K’ points in reciprocal lattice space. Quantum states at K and K’ points are also degenerated in magnetic fields. The eight-fold degenerated zero-mode Landau levels, which were observed in present experiment for *D*_⊥_ = 0, are due to inequivalent quantum states for K and K’ points. Zero-mode Landau level splitting due to a perpendicular electric field is a result of breaking of spatial inversion symmetry. Moreover Landau level calculation based on effective mass approximation indicated that the splitting is due to the valley-splitting; quantum states for K and K’ points are no longer equivalent, and the zero-mode Landau levels show large valley splitting while those levels with larger indices show smaller valley-splitting, which is consistent with our observation.

In AB-stacked tetralayer graphene samples with a single gate electrode, the valley split zero-mode Landau levels with degeneracy of four or less are always observed rather than eight-fold degenerate Landau levels even though a perpendicular electric field is not intentionally applied by using top and bottom electrodes. This is because spatial inversion symmetry is broken owing to the electrostatic potential due to gate induced charges which screens gate electric fields^24^ (see the Supplementary Information).

### Multilayer graphene

The multiple-peak structure in *V*_*g*_ dependence of resistance at zero magnetic field would be a general property of multilayer graphene with four or more layers. In six-layer graphene we found ridge structures similar to those in Fig. 2b,c but with more, which reflected three sets of bilayer-like bands (see the Supplementary Information). These properties of multilayer graphene have not been reported previously. This would be because mobility of the samples was not sufficiently high. Indeed, low mobility samples show a broad peak or broad peaks, and it is difficult to discuss electronic band structure.

### Summary

We have studied transport property of AB-stacked tetralayer graphene samples which are encapsulated with *h*-BN flakes and have top and bottom gate electrodes. We found that, at zero magnetic field, the top and bottom gate voltage dependence of resistance shows non-trivial ridge structures which stem from band structures. By analyzing Landau fan diagrams, positions of some ridge structures were verified to correspond to those of the zero-mode Landau levels of bilayer-like bands. They are eight-fold degenerated at zero perpendicular electric field while they split into two four-fold degenerated levels in presence of perpendicular electric field. Correspondingly, the ridge structures crossed at zero electric field. On the other hand resistance increased by applying perpendicular electric field at the charge neutrality point at zero magnetic field, however, strong insulating behavior was not observed. In AB-stacked six-layer graphene we also found similar ridge structures. The ridges that appear in the top and bottom gate voltage dependence would be a common feature of multilayer graphene with number of layers larger than three.

## Methods

Graphene was prepared by mechanically exfoliating high-quality Kish graphite using adhesive tape. Graphene was encapsulated with high quality *h*-BN flakes. The top gate was several-layer graphene that was deposited on top of the encapsulated graphene. Each sample was patterned into a Hall bar using standard electron beam lithography. Electric resistance was measured using the standard lock-in technique with a low-frequency excitation current whose frequency and amplitude were typically 10 Hz and 1 μA. Magnetic fields were applied by using a superconducting solenoid. Transport measurements were performed at temperature of 4.2 K.

## Electronic supplementary material

Supplementary Information
