# Supplementary material for: Multilayer graphene shows intrinsic resistance peaks in the carrier density dependence
Source: Sci Rep. 2018 Sep 18;8:13992. doi: 10.1038/s41598-018-32214-7 (PMC6143556; doi:10.1038/s41598-018-32214-7)

Supplementary Information

Multilayer graphene shows intrinsic resistance peaks in the carrier density dependence.

Taiki Hirahara[1,] Ryoya Ebisuoka[1], Takushi Oka[1], Tomoaki Nakasuga[1], Shingo Tajima[1], Kenji Watanabe[2], Takashi Taniguchi[2] and Ryuta Yagi[1]

[1] Graduate School of Advanced Sciences of Matter, Hiroshima University, Higashi-Hiroshima, Hiroshima 739-8530, Japan.
[2] National Institute for Materials Science (NIMS), 1-1-1 Namiki, Tsukuba 305-0044, Japan.

Sample fabrication

Thin flakes of *h*-BN and graphene were made by mechanical cleaving with adhesive tape [1]. Graphene was encapsulated by successively piling up flakes of *h*-BN, graphene and *h*-BN on SiO_2 substrates by using a dry-process transferring technique with poly (propylene carbonate) (PPC) films [2]. Several-layer graphene was transferred onto the top of the encapsulated graphene by using the washer method [3]. The sample was patterned by using electron beam lithography (EBL) and plasma etching. The electrical leads were formed using EBL and vacuum deposition of Cr and Au. Electrical contacts between the leads and graphene were attained using the technique described in Ref. [2].

Mobility of Sample

Figure S1 shows gate voltage dependence of electric mobility that was

calculated using $\mu = 1/en_{tot}\rho$. Mobility at $V_{bg} = \pm 50$ V and $V_{tg} = 0$ V is about 40,000 cm^2/Vs.

Checking number of layers and stacking order of tetralayer graphene

Determinations of number of layers and stacking order are crucial in the study of multilayer graphene. They were determined by color contrast of the digitized optical image, Raman G' spectra shape, AFM topography and the Landau fan diagram. Number of layers can be readily identified by analyzing color digit of the optical images of graphene flakes which were exfoliated on the SiO_2 (300 nm thick)/Si (p+ doped) substrate. First, we calibrated relationship between the color and the thickness. The optical micrograph of a representative sample which was used in the calibration is shown in Fig. S2**a**. We checked the thickness of graphene by using the AFM measurements. An example of AFM topography is shown in Fig. S2**b**, which is measured for the square region in Fig. S2**a**. A line scan along the red line is shown in Fig. S2**c**. Step height is given by about 0.33 nm/ layer, which is approximately the same as the inter-layer distance of graphite.

For many graphene samples with identified number of layers, we performed the Raman spectra measurements. Figure S3**a** shows Raman G' band spectra of graphene flakes with 2 to 5 layers. Here, the data were offset and normalized. These approximately reproduce the results of AB-stacked graphene which have been reported by other groups [8-10]. The spectra shown in Fig. S3**a** appeared most frequently in graphene sample prepared from our Kish graphite crystals. Actually, in particular batches of graphite crystals that was used to prepare the sample for the transport experiment, we did not find any graphene flakes that show G' band spectra shapes other than Fig. S3**a.** In other batches of graphite crystals, we found flakes that show Raman G' band spectral shape for ABC-stacking [9-11], as shown in Fig.

S3**b**.

For the sample that has been shown in the main text, we could not perform the Raman measurement because performing the Raman measurements during the fabrication process might contaminate surfaces of graphene or *h*-BN. In addition, after finishing making sample, the Raman measurement is impossible because graphene to be measured is underneath thick graphene and *h*-BN stack serving as the top gate electrode. However, a proof for the AB-stacked tetralayer graphene can be found in the structure of Landau levels. Fig. S4**a** shows the Landau fan diagram of another AB-stacked tetra-layer graphene sample, which was simply formed on a *h*-BN flake and not encapsulated. This sample was identified to be AB-stacked tetralayer graphene by the Raman spectroscopy. The Raman G' band spectra shape indicated AB-stacked tetralayer graphene. Moreover, a map of Raman G band spectral intensity indicated that the region relevant to the transport experiment is uniform as shown in Fig. S4**b**, which ensures no ABC stacks in the region.

The basic structures of Landau level are approximately the same between the abovementioned sample and the samples described in the main text and supplementary: conspicuous energy gaps and zero-mode Landau levels appeared approximately the same positions as shown with blue lines in Figs. S4**a** and S4**c**, (The data displayed in Fig. S4**c** are the same as Figs. 3**c** and **d** in the main text). The Landau level structure reflects the dispersion relation, and it results from the number of layers and stacking order of graphene. Therefore, data which are displayed in the main text are for AB-stacked tetralayer graphene.

In both Figs. S4**a** and S4 **c**, the zero-mode Landau level of light-mass bilayer-like band which is eight-fold degenerate at $D_{\perp} = 0$ (Fig. 3**a** in main text) split into two levels with four-fold degeneracy. We found that the gap

width in n_{tot} between the split of zero-mode Landau levels is slightly different between Figs. S4**a** and S4**c**. This originates from difference in the effective perpendicular electric fields. In case of Fig. S4**c**, perpendicular electric fields (electric flux density) with a fixed magnitude are applied by using top and bottom gate electrode. On the other hand, in case of Fig. S4, the sample has a single bottom gate electrode, so that magnitude of the perpendicular electric field (electric flux density) varies with gate voltages. Carriers that are induced by gate voltage are expected to distribute in graphene over a characteristic screening length.

Reproducibility of the ridge structures at zero magnetic field

Figure S5 **a** shows n_{bg} and n_{tg} dependence of resistivity ρ, which was measured in a different AB-stacked tetralayer graphene sample (Sample B). Figure S5**b** shows derivative of rho with respect to n_{bg}. Fig. S5**c** is a replot of the same data as Fig. 2**b** in the main text (Sample A). A measured region for Fig. S5**c** is indicated with a yellow square in Fig. S5b. As seen in the figures, ridges **a**, **b** and **c** were also observed in the both samples. The reproducibility of the ridge structures ensures that the structures originated from the intrinsic nature of tetralayer graphene. In addition, new ridges **e** and **f** are seen in Fig. S5**a** and **b**. The ridges **e** and **f** would arise from band bottoms of the heavy-mass bilayer-like band. Figure S6**a** and **b** show replots of Fig. S5**a** and **c** as a function of n_{tot} and $D_{\perp}$.

Results in six-layer graphene

Figure S7**a** and **b** show similar plot as Fig. 3**b** and 3**c** in the main text for a AB-stacked six-layer graphene sample. The resistance ridge structures that vary with n_{tg} and n_{bg} are also seen, as in the case of tetralayer graphene.

Panel **c** shows **a** derivative of resistance with respect to $D_\perp$. The ridge structure is more complicated than that of the tetralayer. Formation of energy gap similar to the light-mass bilayer-like bands in AB-stacked tetralayer graphene is clearly visible as indicated by dashed lines which show a crossing feature. Here, we do not go into detailed band structures which will be reported elsewhere, but zero-mode Landau levels appear at the position of the intrinsic resistance peaks (not shown).

Band structure of tetralayer graphene

We calculated the energy gap in AB-stacked tetralayer graphene using the standard Hamiltonian for effective mass approximation [4, 5]. Koshino has shown that Hamiltonian H is decomposed into submatrices by a unitary transformation as

$$H = \begin{pmatrix} H_b & H_c \\ H_c & H_B \end{pmatrix},$$

(SF.1)

Here submatirces H_b and H_B are those for the light-mass and heavy-mass bilayer-like bands, respectively, and are given by

$$H_b = \begin{pmatrix} \lambda_+^2 U_1 + \lambda_-^2 U_3 - \lambda\gamma_2 & v\pi^\dagger & -\lambda_b v_4 \pi^\dagger & \lambda_b v_3 \pi \\ v\pi & \lambda_+^2 U_1 + \lambda_-^2 U_3 + \Delta' - \lambda\gamma_5 & \lambda_b \gamma_1 & -\lambda_b v_4 \pi^\dagger \\ -\lambda_b v_4 \pi & \lambda_b \gamma_1 & \lambda_+^2 U_4 + \lambda_-^2 U_2 + \Delta' - \lambda\gamma_5 & v\pi^\dagger \\ \lambda_b v_3 \pi^\dagger & -\lambda_b v_4 \pi & v\pi & \lambda_+^2 U_4 + \lambda_-^2 U_2 - \lambda\gamma_2 \end{pmatrix},$$

(SF.2)

$$H_B = \begin{pmatrix} \lambda_+^2 U_3 + \lambda_-^2 U_1 + \lambda\gamma_2 & v\pi^\dagger & -\lambda_B v_4 \pi^\dagger & \lambda_B v_3 \pi \\ v\pi & \lambda_+^2 U_3 + \lambda_-^2 U_1 + \Delta' + \lambda\gamma_5 & \lambda_B \gamma_1 & -\lambda_B v_4 \pi^\dagger \\ -\lambda_B v_4 \pi & \lambda_B \gamma_1 & \lambda_+^2 U_2 + \lambda_-^2 U_4 + \Delta' + \lambda\gamma_5 & v\pi^\dagger \\ \lambda_B v_3 \pi^\dagger & -\lambda_B v_4 \pi & v\pi & \lambda_+^2 U_2 + \lambda_-^2 U_4 + \lambda\gamma_2 \end{pmatrix}. \tag{SF.3}$$

Here $\pi = \hbar(k_x + ik_y)$, $v = (\sqrt{3}/2)a\gamma_0/\hbar$, and $v_1 = (\sqrt{3}/2)a\gamma_1/\hbar$, where a = 0.246 nm is the lattice constant of the graphene, and γ_i (i = 0,1,2,...,5) an Δ' are SWMcC (Slonczewski-Weiss-McClure) parameters [6, 7]. $\lambda_\pm$, λ_b, λ_B, λ are constants given by

$$\lambda_\pm = \sqrt{\frac{5\pm\sqrt{5}}{10}}, \tag{SF.4}$$

$$\lambda_b = (-1+\sqrt{5})/2, \tag{SF.5}$$

$$\lambda_B = (1+\sqrt{5})/2, \tag{SF.6}$$

$$\lambda = 1/\sqrt{5}. \tag{SF.7}$$

We added to the diagonal element of the Hamiltonian matrix the potential energy for each layer U_i , where i is an index of layer number. The submatirix H_c, which couples two bilayer-like bands, is given by

$$H_c = \begin{pmatrix} \lambda(U_1 - U_3 + \gamma_2/2) & 0 & 0 & 0 \\ 0 & \lambda(U_1 - U_3 + \gamma_5/2) & 0 & 0 \\ 0 & 0 & \lambda(U_2 - U_4 - \gamma_5/2) & 0 \\ 0 & 0 & 0 & \lambda(U_2 - U_4 - \gamma_2/2) \end{pmatrix}, \tag{SF.8}$$

If H_c is ignored, the energies of the bottoms of the conduction and valence band are analytically calculated to be

$$\varepsilon_{k=0}^{light} = \begin{cases} \lambda_+^2 U_1 + \lambda_-^2 U_3 - p\gamma_2 \\ \lambda_+^2 U_4 + \lambda_-^2 U_2 - p\gamma_2, \end{cases} \tag{SF9}$$

and

$$\varepsilon_{k=0}^{heavy} = \begin{cases} \lambda_+^2 U_3 + \lambda_-^2 U_1 + p\gamma_2 \\ \lambda_+^2 U_2 + \lambda_-^2 U_4 + p\gamma_2. \end{cases}$$

(SF10)

Therefore, energy gaps at $k = 0$ are simply calculated to be

$$\Delta\varepsilon_{k=0}^{light} = |\lambda_+^2(U_1 - U_4) + \lambda_-^2(U_3 - U_2)|,$$

(SF11)

and

$$\Delta\varepsilon_{k=0}^{heavy} = |\lambda_+^2(U_3 - U_2) + \lambda_-^2(U_1 - U_4)|.$$

(SF12)

Here, we consider a particular case that the potential energy is antisymmetric, *i.e.*, $U_1 = -U_4$ and $U_2 = -U_3$. Then the differences of the potential energy $U_3 - U_2$ and $U_1 - U_4$ can be written by using the two parameters $\Delta U = U_1 - U_4$ and r as

$$U_2 - U_3 = r(U_1 - U_4) = r\Delta U.$$

(SF13)

Then,

$$\Delta\varepsilon_{k=0}^{light} = (\lambda_+^2 - r\lambda_-^2)|\Delta U|,$$

(SF14)

And

$$\Delta\varepsilon_{k=0}^{heavy} = (\lambda_-^2 - r\lambda_+^2)|\Delta U|.$$

(SF15)

In a weak external field, the gaps are approximately proportional to $|D_\perp|$ because $|\Delta U|$ is roughly proportional to $|D_\perp|$. The light-mass bilayer-like band is expected to have a larger energy gap than the heavy-mass bilayer-like band.

Landau levels in tetralayer graphene.

Landau levels of tetralayer graphene are valley-degenerated because the system has a spatial inversion symmetry. The spatial inversion symmetry can be broken by applying electric fields by using gate voltages and giving potential variations in each layer. Then the valley degeneracy of the Landau levels should be lifted. Here we show this by a simple numerical calculation of the energy spectra of tetralayer graphene in a magnetic field. We calculated the energy spectra in the frame of effective mass approximation that considers all the SWMcC parameters [4]. We expanded the wave functions with the Landau functions, and the electric potential at each layer was added to the diagonal element of the Hamiltonian matrix. Figure S8**a** shows energy spectra of tetralayer graphene in the absence of external potential variation. The SWMcC parameters of this calculation were taken to be the same as those of graphite, *i.e.*, $\gamma_0 = 3.16$ eV, $\gamma_1 = 0.39$ eV, $\gamma_2 = -0.02$ eV, $\gamma_3 = 0.31$ eV, $\gamma_4 = 0.44$ eV, $\gamma_5 = 0.038$ eV, and $\Delta' = \Delta - \gamma_2 + \gamma_5 = 0.037$ eV. Spectra for the K valley and K' valley are identical so that each line is four-fold degenerated because of the degeneracy in spin and valley degrees of freedom. Next we show the result in the presence of potential variation in graphene. We assumed a model potential. From the top layer to the bottom layer, the electric potential energy was -0.01, -0.005, $+0.005$, and $+0.01$ eV. This breaks spatial inversion symmetry. Figure S8**b** shows results of the calculation. Red and black lines are energy eigenvalues for the K and the K' valleys, respectively. It is seen that energy spectra of K and K' valleys differ slightly. In particular, zero-mode Landau levels showed splitting, which corresponds to forming an energy gap at the bottoms of the conduction and valence bands of the bilayer-like band. Valley splitting can be seen in the energy spectra of trilayer graphene even in the absence of external potential variation [4].

On insulating behavior at $D_\perp = 0$

In four different samples of AB-stacked tetralayer graphene, we have not observed the strong insulating behavior that has been reported in Ref. 18 in the main text. The study attributed the insulating behavior to possible staggered potential. We estimated maximum possible amplitude of the staggered potential at the neutrality point by model calculations. With the increasing strength of the staggered potential, the zero-mode Landau levels split as shown by the arrows in Fig. S9**a**. In our experiments, splitting of the zero-mode Landau level was not observed for $D_{\perp} = 0$. (Fig. S9**b**.) This would indicate that the staggered potential is absent or its amplitude is significantly small. A model calculation indicates that if the staggered potential amplitude is less than about ~0.0005 eV, splitting of zero-mode Landau levels is within the width of the zero-mode Landau levels in the experimental fan diagram. Therefore, presence of staggered potential does not contradict with our experiment as long as the amplitude is sufficiently small. However, to be discussed in the next section, energy gap does not form between the conduction and valence bands for this magnitude of staggered potential at $D_{\perp} = 0$.

The origin of the difference between our results and Ref. 18 in the main text is currently unknown. This might be sought in the experimental setup: graphene is suspended in Ref. 18 in the main text, while it is encapsulated in the present study.

Dispersion relation in presence of perpendicular electric field.

Next we discuss the dispersion relation of electronic band structure at zero magnetic fields. Variations of the band structure as a function of a perpendicular electric field were calculated numerically using the effective mass approximation with Slonczewski-Weiss-McClure parameters of

graphite. Figure S10 shows results in the presence of electric fields which are created by charges induced by top gate and the bottom gate voltages. Here, n_t and n_b are the charges induced by top and bottom gate voltages, respectively, and $p = \hbar k_x$. We assumed characteristic screening length of 0.43 nm and permittivity of graphene of 2. Figure S11 shows enlargement plots near $E/\gamma_0 = 0$.

In the absence of perpendicular electric field, *i.e.*, $n_t = 0$ and $n_b = 0$, the calculated dispersion relation is essentially the same as that reported in [4, 5]. Tetalayer graphene has two sets of bilayer-like bands. Coupling between these bands tends to open an energy gap in the vicinity of E= 0. However, because of the trigonal warping, energy gap closes partially, and conduction band and valence band touches or overlaps at very small regions in k-space [4, 5] (Point X in Fig. S10).

The perpendicular electric field varies the dispersion relations, as seen in Figure S10. The most conspicuous change is that energy gaps are formed between band A and B, and between band C and D. Bottoms of the bands are flattened, which increases band masses. In addition, mini-Dirac cones appear near $E/\gamma_0 = 0$. Even with increasing $|D_\perp| = |e(n_b - n_t)|$, the valence band and the conduction band remain touching, as seen in Figs. S10 and S11. The magnitude of group velocity near the touching point is seen to become larger with increasing $|D_\perp|$.

The variation of the dispersion in the vicinity of E= 0 could result in considerable change in the resistivity. In addition, the variation of the resistivity could originate from formation of energy gap between conduction and valence band. Indeed, the band structure in the vicinity of $E = 0$ is strongly dependent on γ_3 in the SWMcC parameters, which describes the trigonal warping. In the case of $\gamma_3 = 0.28$ eV, which is slightly smaller than the parameter of graphite, an energy gap opens between bands B and C in

the presence of perpendicular electric field as shown in Figure S12, while the conduction and valence bands are touching or overlapped at $|D_{\perp}| = 0$.

Even for SWMcC parameter of graphite, opening the energy gap via the perpendicular electric field is possible if the staggered potential is present. Figures S13 and S14 show the results of for $\gamma_3 = 0.3$ eV and with staggered potentials with amplitude of 0.5 and 1 meV, respectively. The perpendicular electric field opens energy gaps with a few mil electron volts between bands B and C, while, at zero electric field, no energy gap open.

The simplified band structure described in the main text would have the characteristic features of the above band models in terms of formation of the energy gap.

Thickness of *h*-BN and dielectric breakdown

In the sample fabrication process, we have not measured the thickness of the *h*-BN flakes of the top gate insulator by using atomic force microscopy (AFM) to avoid contamination. The thickness of top *h*-BN underneath the top gate graphene is difficult to measure by using AFM after finishing making samples because of the sample structure. We estimated the thickness of the *h*-BN flake by using two different methods other than the AFM measurement. The first method is to analyze the color of the *h*-BN flakes. We calibrated the relationship between the thickness that was measured by AFM and the intensity of the RGB-signal of the digitized optical micrograph of *h*-BN flakes, which were exfoliated on the SiO_2/Si substrate. The thicknesses of the *h*-BN flakes for the top gate insulator were estimated to be 15 ± 4 nm for sample A (data are presented in the main text), and 65 ± 4 nm for sample B (data are presented in Figs. S2**a**). The thicknesses of the bottom *h*-BN flakes were both about 10 nm for samples A and B.

The second method is to use the ratio of capacitances C_{tg}/C_{bg}. According to the classical theory of electromagnetism, $C_{tg} = \epsilon_0 \epsilon_{hBN}/d_{\hbar BN}$ and $C_{bg} = \epsilon_0 \epsilon_{SiO2} /d_{SiO2}$. Here, $d_{\hbar BN}$ and d_{SiO2} are the thicknesses of the gate insulators, *i.e*, *h*-BN and SiO_2, respectively. ϵ_0 is the dielectric constant of vacuum. $\epsilon_{\hbar BN}$ and ϵ_{SiO2} are the relative permittivity of *h*-BN and SiO_2, respectively. $\epsilon_{\hbar BN}$ is about 3.9. $\epsilon_{\hbar BN}$ is reported to be 3-4 [12, 13], which is close to the value of $\epsilon_{\hbar BN}$. The ratio C_{tg}/C_{bg} was estimated from the condition of $n_{tot} = 0$ to be 13.4 and 3.82 for samples A and B, respectively. If we ignore the thickness of bottom *h*-BN (~ 10nm) which is much thinner than that of SiO_2 (d_{SiO2}= 300nm), $d_{\hbar BN}$ is estimated to be 23 and 89 nm for samples A and B, respectively.

The dielectric breakdown in mechanically exfoliated *h*-BN is reported to occur at about 7 MV/cm [14-16]. Breakdown voltages for the top gate are estimated to be 16 V and 55 V in samples A and B. Therefore experiments were done well below the breakdown voltages.

What happens when the gate voltage exceeds dielectric breakdown limit? Figure S15 shows an example of the dielectric breakdown in a AB-stacked six-layer graphene sample (the sample is different from that for Fig. S7). In this sample, there was a considerable leak current through the top gate electrode for $|V_{tg}|$>5V, and therefore, the ridge structure appearing at $n_{tot} = 0$ is curved. This clearly indicates that top gate does not operate normally for $|V_{tg}|$>5V, and is strikingly different from the results in the AB-stacked six-layer sample which showed a negligible gate leak current. From these results, we can conclude the effects of dielectric breakdown

cannot be observed in the data displayed in the main text, and Figs. S2**a** and **c**.

References

[1] Novoselov, K.S. *et al.*, Two-dimensional gas of massless Dirac fermions in graphene, Nature **438**, 197 (2005).

[2] Wang, L. *et al.*, One-dimensional electrical contact to a two-dimensional material, Science **342**, 614 (2013).

[3] Taychatanapat, T., Watanabe, K., Taniguchi, T. & Jarillo-Herrero, P., Quantum Hall efect and Landau-level crossing of Dirac fermions in trilayer graphene, Nature PhyS8, 621 (2011).

[4] Koshino, M. & McCann, E., Landau level spectra and the quantum Hall effect of multilayer graphene, Phys. Rev. B **83**, 165443 (2011).

[5] Koshino, M. & Ando, T., Orbital diamagnetism in multilayer graphenes, Phys. Rev. B **76**, 085425 (2007).

[6] Slonczewski, J.C. & Weiss, P.R., Band structure of graphite, Phys. Rev. **109**, 272 (1958).

[7] McClure, J.W., Band structure of graphite and de Haas-van Alphen effect, Phys. Rev. **108**, 612 (1957).

[8] Ferrari, A.C. *et al.*, Raman spectrum of graphene and graphene layers, Phys. Rev. Lett. **97**, 187401 (2006).

[9] Lui, C. H. *et al.*, Imaging stacking order in few-layer graphene, Nano Lett. **11**, 164 (2011).

[10] Nguyen, T. A., Lee, J. U., Yoon, D. & Cheong, H., Excitation energy dependent Raman signatures of ABA- and ABC-stacked few-layer graphene, Sci. Rep. **4**, 4630 (2014).

[11] Hao, Y. F. *et al.*, Probing layer number and stacking order of few-layer graphene by Raman spectroscopy, Small, **6**, 195 (2010).

[12] Young, A. F. *et al.*, Electronic compressibility of layer-polarized bilayer graphene, Phys. Rev. B **85**, 235458 (2012).

[13] Laturia, A., Van de Put, M. L. & Vandenberghe, W. G., Dielectric properties of hexagonal boron nitride and transition metal dichalcogenides, 2D Mater. **2**, 235458 (2018).

[14] Hattori, Y., Taniguchi, T., Watanabe, K. & Nagashio, K., Layer-by-layer dielectric breakdown of hexagonal boron nitride, ACS Nano **9**, 916 (2015).
[15] Lee, G. H. *et al.*, Electron tunneling through atomically flat and ultrathin hexagonal boron nitride, Appl. Phys. Lett. **99**, 243114 (2011).
[16] Britnell, L. *et al.*, Electron tunneling through ultrathin boron nitride crystalline barriers, Nano Lett. **12**, 1707 (2012).

Figure Captions

Figure S1 The mobility of the sample. |

V_{bg}-dependence of mobility $\mu = 1/n_{bg}e\rho$ of the tetralayer graphene sample. T= 4.2 K, and B= 0. V_{tg}= 0 V.

Figure S2 Number of layers for graphene flakes. |

a An optical micrograph of a graphene flake consisting of regions with different thicknesses. Numbers show number of layers. **b** AFM Topography for the region displayed with yellow square in panel a. **c** A topographic line scan along the line indicated in panel **b**.

Figure S3 The spectral shapes of Raman G' band for a few layer graphene. |

a Raman spectra of G' band for AB-stacked graphene. The intensity of the spectra is normalized and offset . The spectral shapes of the G' band varied systematically from 2 to 5 layers. **b** Raman spectra of G' band spectra for ABC-stacked trilayer graphene and ABC-stacked tetralayer graphene.

Figure S4 The Landau fan diagrams of AB-stacked tetralayer graphene |

a Landau fan diagram of the AB-stacked tetra-layer graphene sample which is different from those described in the text. T= 4.2 K. Graphene is not encapsulated but placed simply on h-BN. Some energy gaps between Landau levels are indicated with blue lines. The red arrows indicate zero-mode Landau levels. The white dashed lines indicate a measure of filling factors ν. **b** A map of the Raman spectral intensity of G band of the same sample. The square shows the region relevant to the magnetotransport measurements. At any point in the square region, the Raman spectral shapes for G' band spectra were approximately the same as the AB-stacked tetralayer graphene shown in Figure S3a. **c** Landau fan diagrams, which is the same as Fig. 3**c** and **d** in the main text, are shown to compare with panel **a**. Some energy gaps between Landau levels are indicated with blue lines. Red arrows indicate zero-mode Landau levels. Landau level structure is approximately the

same as that of panel **a**.

Figure S5 Reproducibility of n_{tg} and n_{bg} dependence of resistance at zero magnetic field. |

a n_{bg} and n_{tg} dependence of resistivity measured in another sample. **b** Derivative of resistivity with respect to n_{bg}. T= 4.2 K. The resistance ridges arising from band structure are shown with white dashed lines. The ridge structures a b c and d were observed. Ridges e and f are newly observed structure in the hole regime that would correspond to ridges b and c in the electron regime. **c** The same as Fig. 2**b** in the main text. The rectangular regime indicated by yellow lines in panel **b** corresponds to the regime for this measurement. The positions of ridges **e** and **f** are indicated.

Figure S6 Reproducibility of n_{tot} and n_{tg} dependence of resistance at zero magnetic field. |

a The replot of Fig. S5**a** as a function of n_{tot} and $D_{\perp}$. **b** The replot of Fig S5**c** as a function of n_{tot} and $D_{\perp}$.

Figure S7 The results in AB-stacked six-layer graphene. |

a Top and bottom gate voltage dependence of resistance (R) at zero magnetic field. Gate voltages were converted to the carrier densities n_{tg} and n_{bg} associated with the gate voltages. T= 4.2 K. **b** Replot as a function of n_{tot} and $D_{\perp}$. **c** Derivative of R with respect to $D_{\perp}$.

Figure S8 The Landau levels in tetralayer graphene. |

a Landau levels in the absence of external potential variation. Results for K and K' valleys are degenerated. **b** Landau levels in the presence of external potential variation. Red and black lines are those for the K valley and the K' valley, respectively. The SWMcC parameters for these calculations were the same as those of graphite. **c** The definition of SWMcC parameters.

Figure S9 The effect of staggered potential on Landau fan diagram |

a Calculated Landau fan diagrams for different values of staggered potential amplitude. Density of states was plotted with respect to total carrier n_{tot}. An inset illustrates the definition of staggered potential. The position of zero-mode Landau level of light-mass bilayer-like band is shown by arrows. The zero-mode Landau levels split for $u \neq 0$. SWMcC parameters for these calculations are $\gamma_0 = 3.16$ eV, $\gamma_1 = 0.39$ eV, $\gamma_2 = -0.02$ eV, $\gamma_3 = 0.3$ eV, $\gamma_4 = 0.04$ eV, $\gamma_5 = 0.038$ eV, and $\Delta_p = 0.037$ eV. Bottom inset shows a schematic diagram of the staggered potential in each layer. **b** Experimental Landau fan diagram of AB-stacked tetra-layer graphene at $D_\perp = 0$. The width of the zero-mode Landau level is smaller than that of $u = 0.001$ eV. One can estimate that staggered potential in actual sample is less than about 0.0005 eV.

Figure S10 Dispersion relation of tetralayer graphene with a perpendicular electric field. 1 |

Numerically calculated dispersion relation of AB-stacked tetralayer graphene with a perpendicular electric field applied by using top and bottom gate electrodes. n_t and n_b are charges induced by top and bottom electrodes. SWMcC parameters were taken the same as graphite: $\gamma_0 = 3.16$ eV, $\gamma_1 = 0.39$ eV, $\gamma_2 = -0.02$ eV, $\gamma_3 = 0.3$ eV, $\gamma_4 = 0.04$ eV, $\gamma_5 = 0.038$ eV, and $\Delta_p = 0.037$ eV. Form the left to the right, n_t and n_b were varied as $n_t = -n_b = 0,\ 1\times 10^{12}$, 2×10^{12} , 3×10^{12} cm^{-2}. X indicates a point where the conduction and valence bands touch or overlap. Bands B and C touch near $E/\gamma_0 = 0$.

Figure S11 Dispersion relation of tetralayer graphene with a perpendicular electric field. 2 |

Enlargement of Fig. S10 near $E/\gamma_0 = 0$.

Figure S12 Dispersion relation of tetralayer graphene with a perpendicular electric

field and with smaller γ_3 |

Dispersion relation which was calculated for $\gamma_3 =$ 0.28 eV. Other parameters are the same as those for Figs. S10 and S11.

Figure S13 Dispersion relation of tetralayer graphene with a perpendicular electric field and with staggered potential 1 |

Dispersion relation which was calculated for $u =$ 0.5 meV. Other parameters are the same as those for Figs. S10 and S11.

Figure S14 Dispersion relation of tetralayer graphene with a perpendicular electric field and with staggered potential 2 |

Dispersion relation which was for u = 1 meV. Other parameters are the same as those for Figs. S10 and S11.

Figure S15 Dielectric breakdown in gate voltage dependence of resistance. |

Top and bottom gate voltage dependence of resistance in an AB-stacked six-layer graphene sample which shows a dielectric break down at about $|V_{tg}|$=5 V. Measured sample is different from that six-layer graphene whose result of experiment is presented in Figs. S3**a** - **c**.

Fig. S1

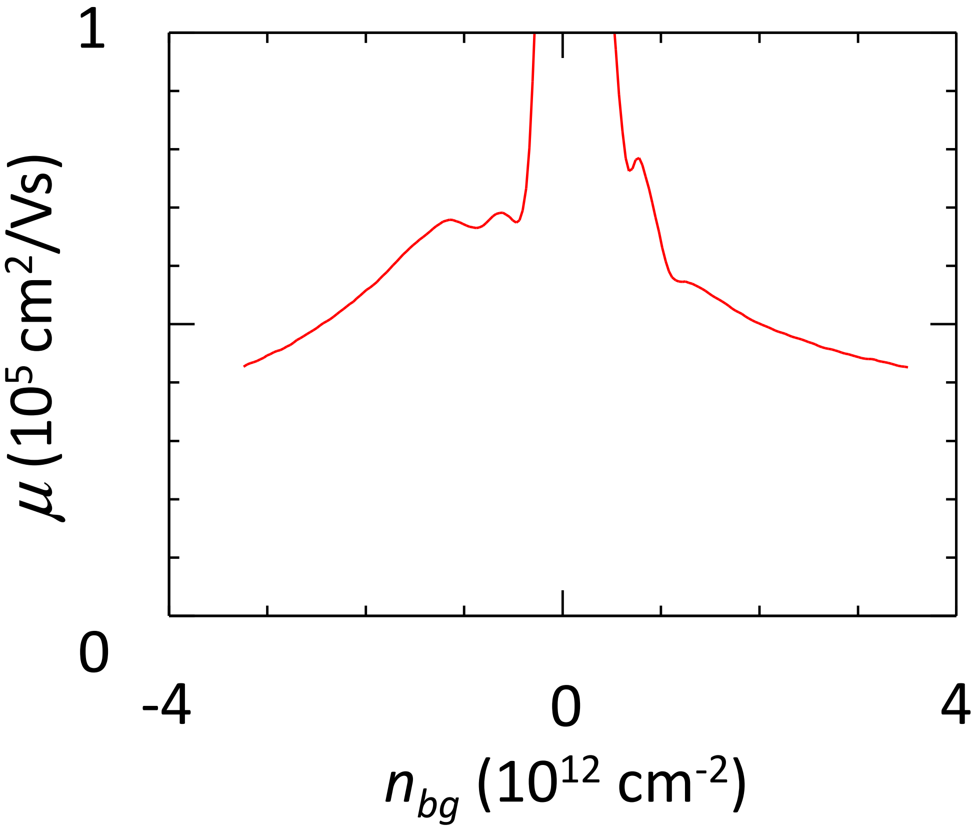

Fig. S2

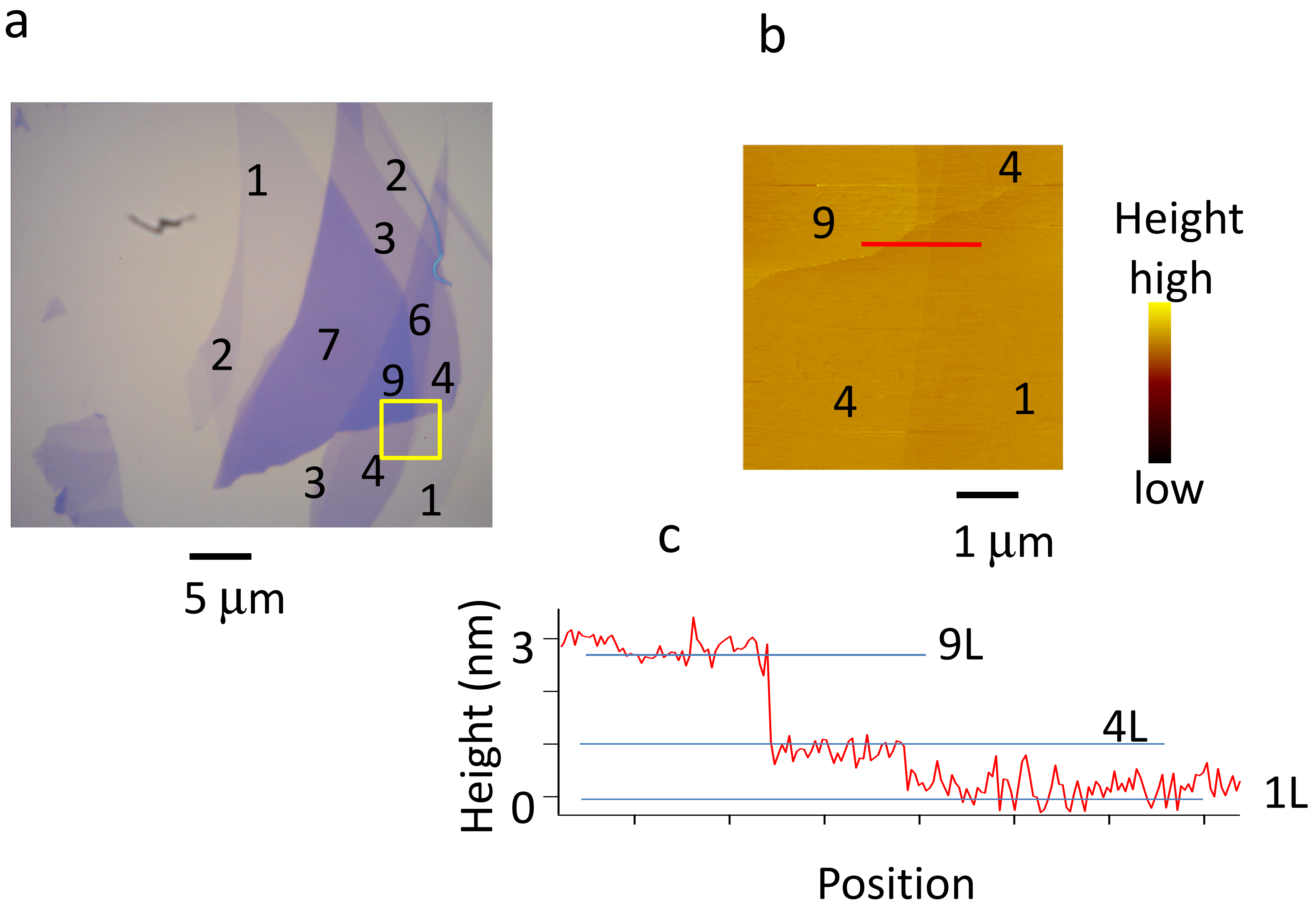
a
1
2
3
6
7
2
9
4
3
4
1
5 μm
b
4
9
4
1
Height
high
low
1 μm
c
Height (nm)
3
0
9L
4L
1L
Position

Fig. S3

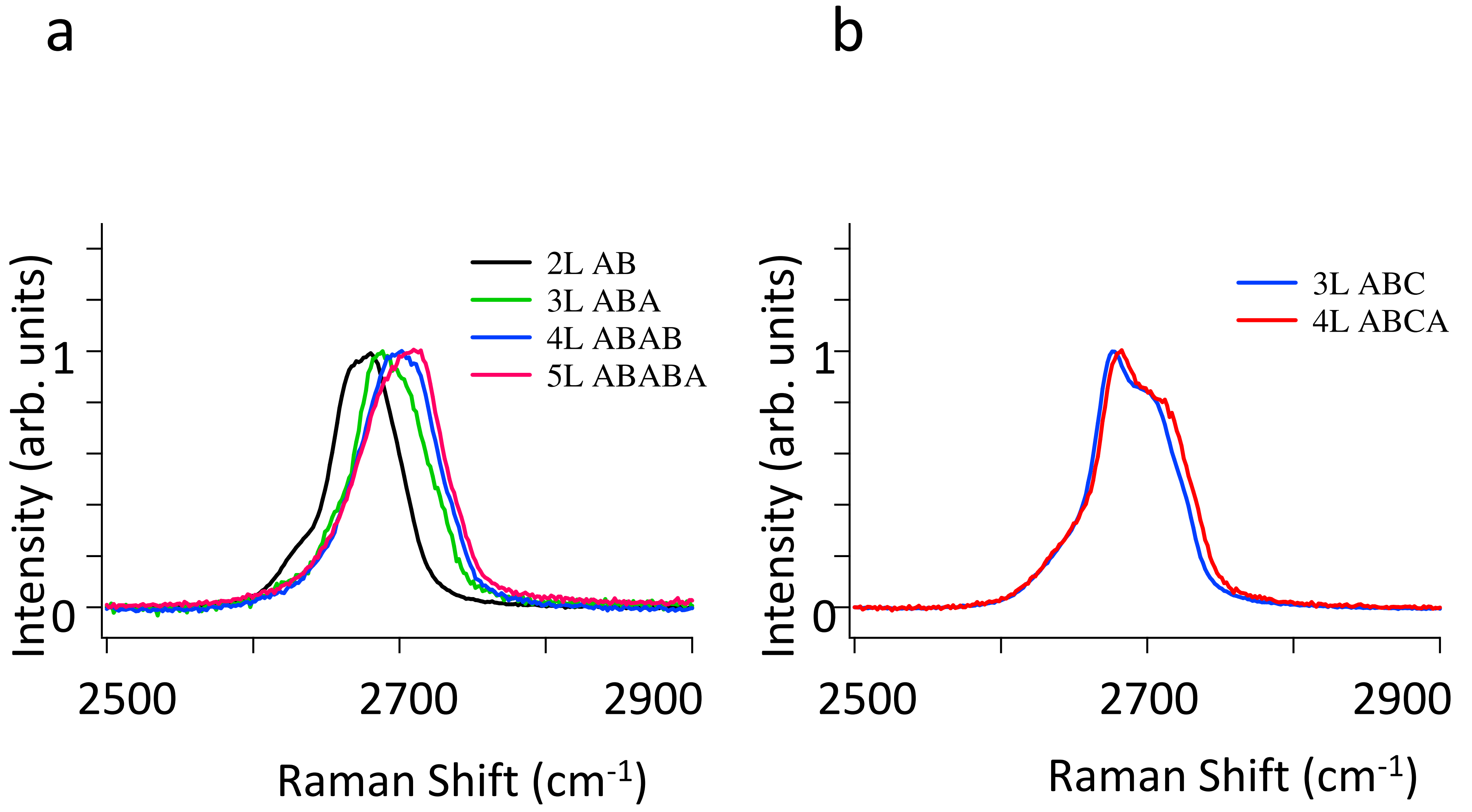

a
b
Intensity (arb. units)
Raman Shift (cm-1)
2500
2700
2900
0
1
2L AB
3L ABA
4L ABAB
5L ABABA
3L ABC
4L ABCA

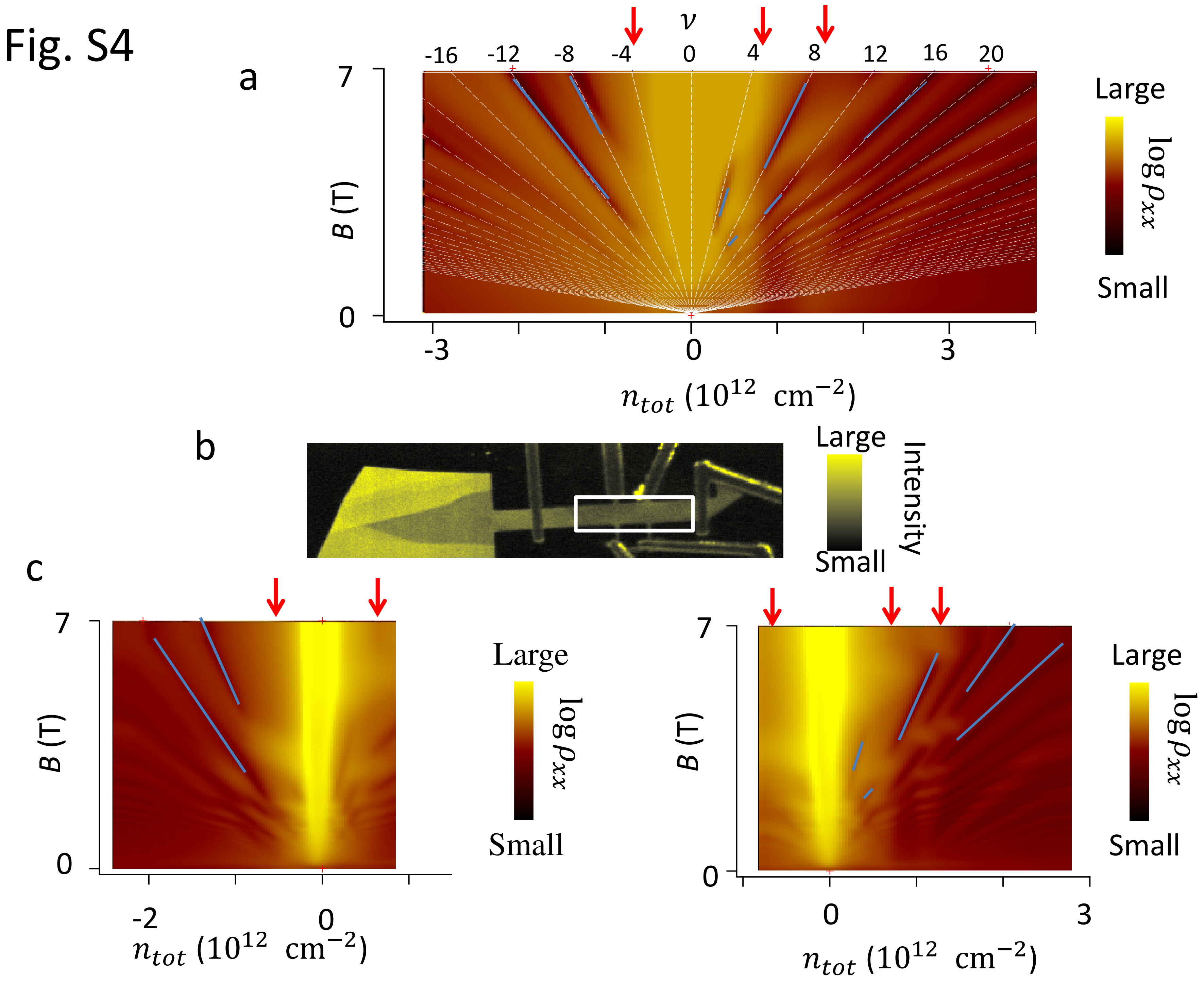
Fig. S4
a
ν
-16 -12 -8 -4 0 4 8 12 16 20
7
0
B (T)
-3 0 3
n_{tot} (10^{12} cm^{-2})
Large
log ρ_{xx}
Small
b
Large
Intensity
Small
c
7
0
B (T)
-2 0
n_{tot} (10^{12} cm^{-2})
Large
log ρ_{xx}
Small
7
0
B (T)
0 3
n_{tot} (10^{12} cm^{-2})
Large
log ρ_{xx}
Small

Fig. S5

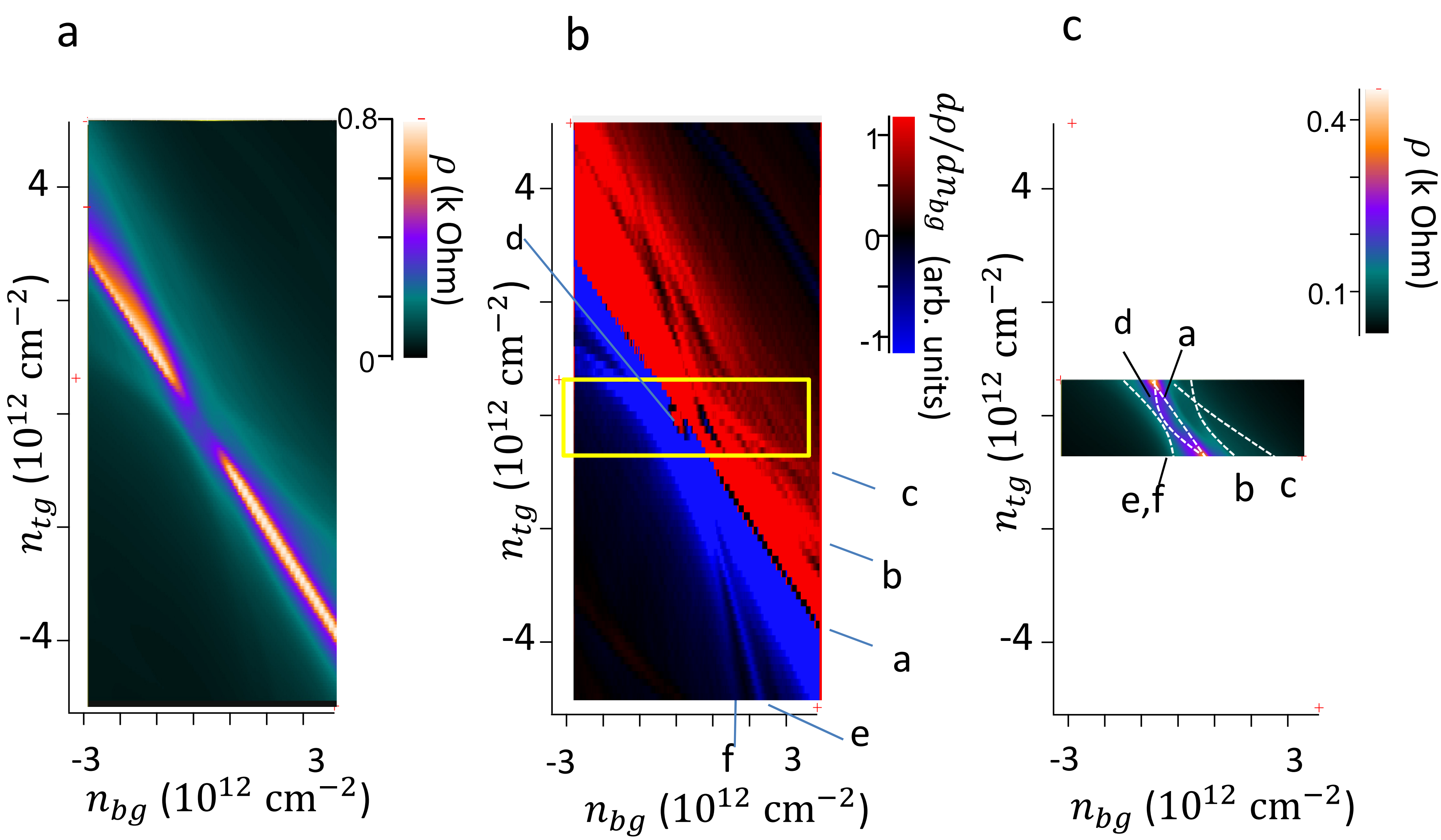

a
b
c
0.8
0
ρ (k Ohm)
$d\rho/dn_{bg}$ (arb. units)
1
0
-1
0.4
0.1
4
-4
-3
3
n_{tg} (10^{12} cm^{-2})
n_{bg} (10^{12} cm^{-2})
d
a
b
c
e
f
e,f

Fig. S6

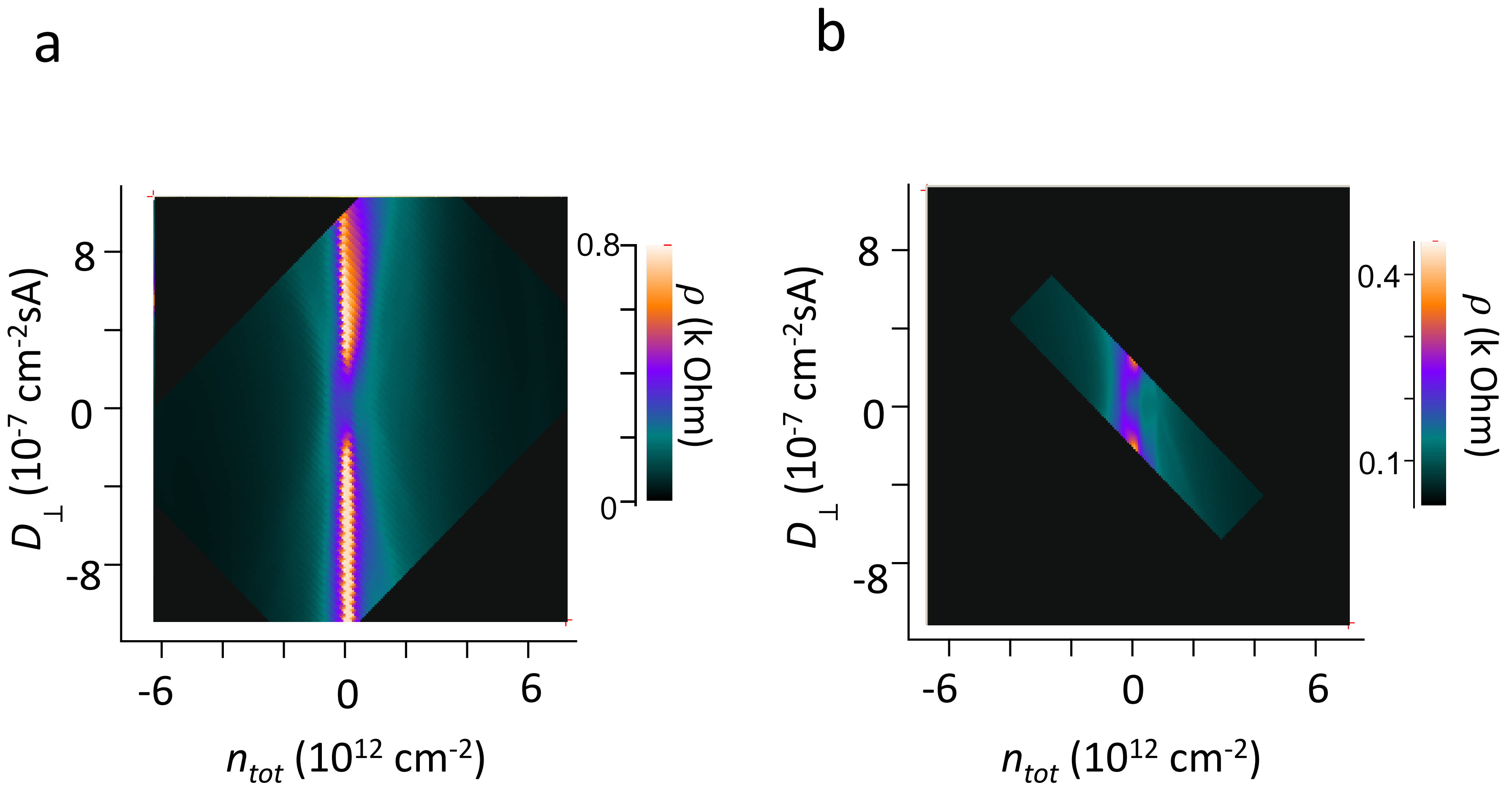

Fig. S7

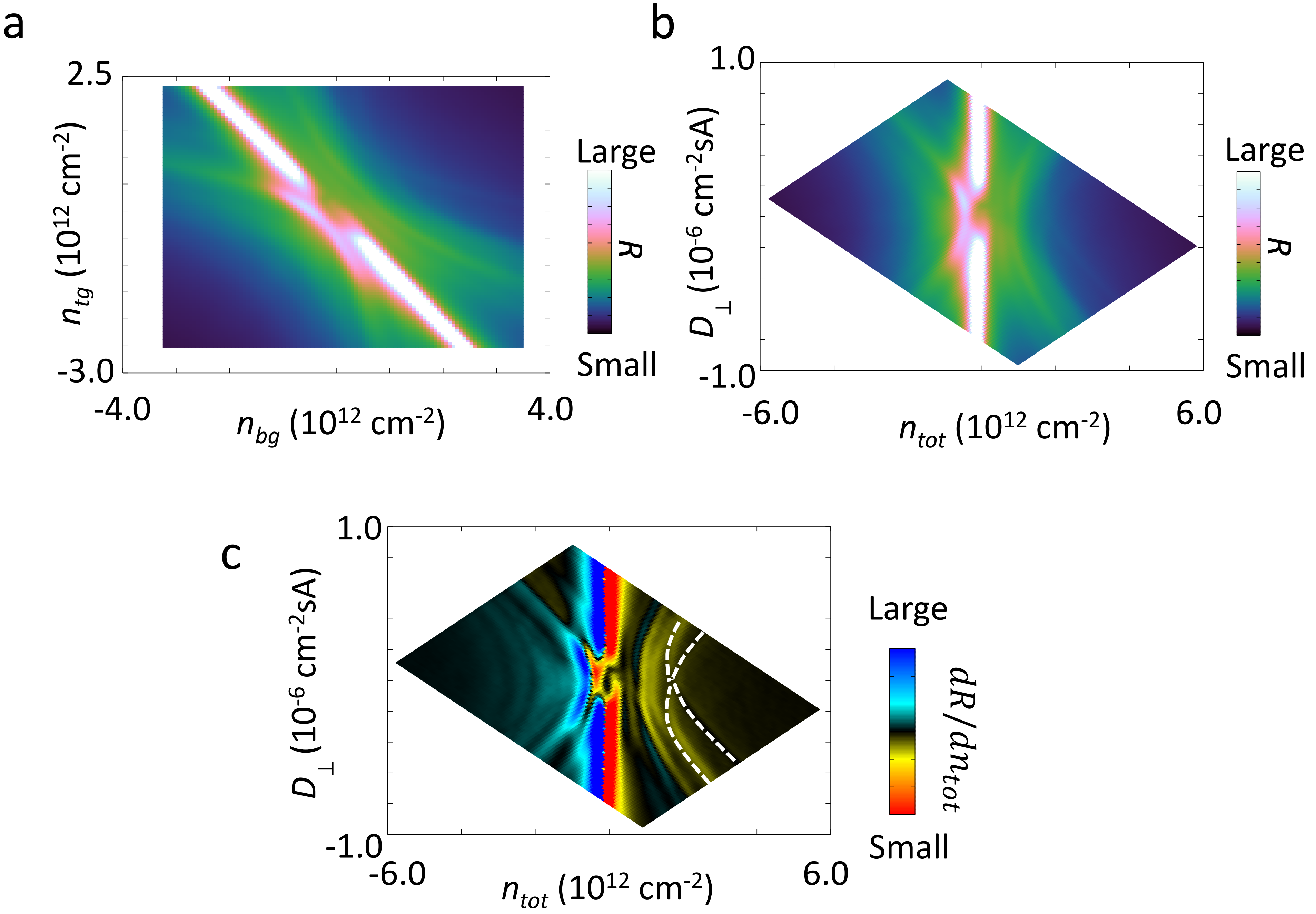
a
b
c
2.5
-3.0
-4.0
4.0
n_{tg} (10^{12} cm^{-2})
n_{bg} (10^{12} cm^{-2})
Large
Small
R
1.0
-1.0
-6.0
6.0
$D_{\perp}$ (10^{-6} cm^{-2}sA)
n_{tot} (10^{12} cm^{-2})
dR/dn_{tot}

Fig. S8

a

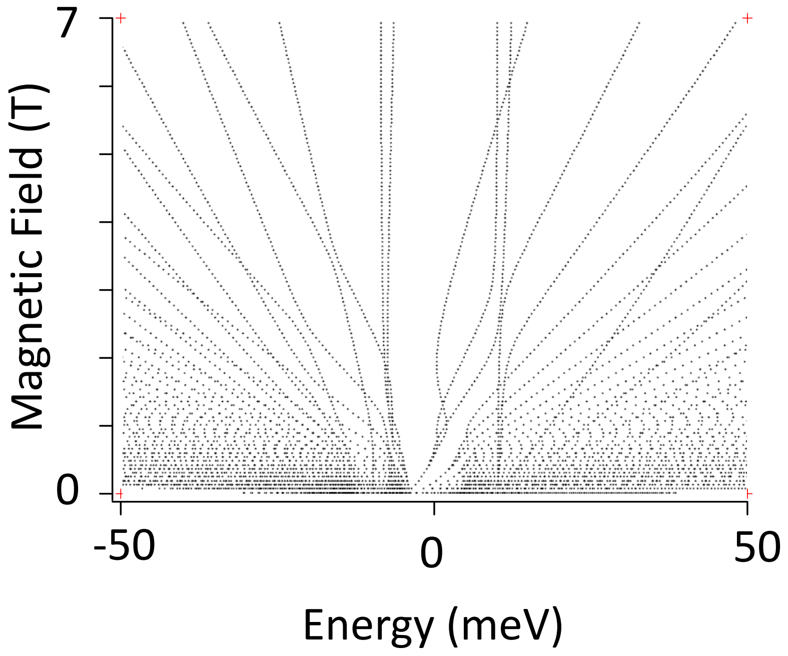

7
0
Magnetic Field (T)
-50
0
50
Energy (meV)

b

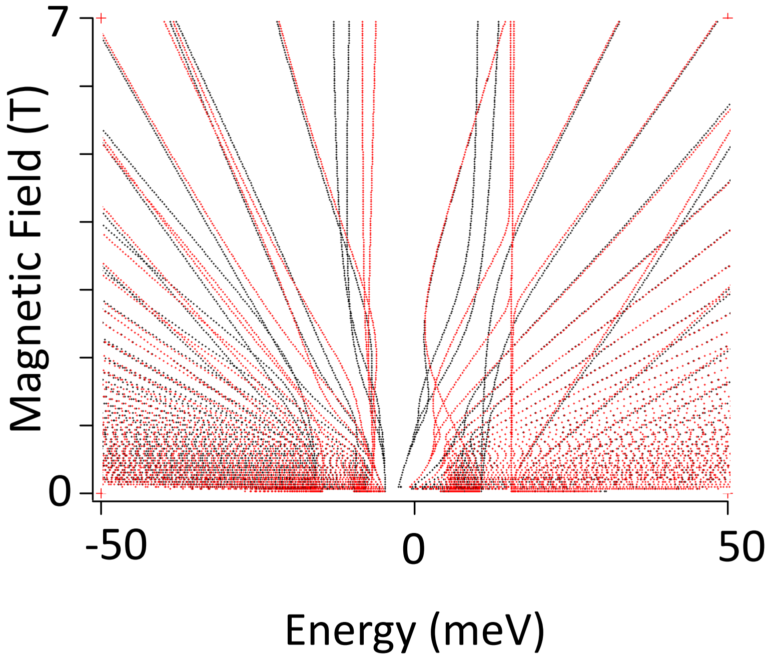

7
0
Magnetic Field (T)
-50
0
50
Energy (meV)

c

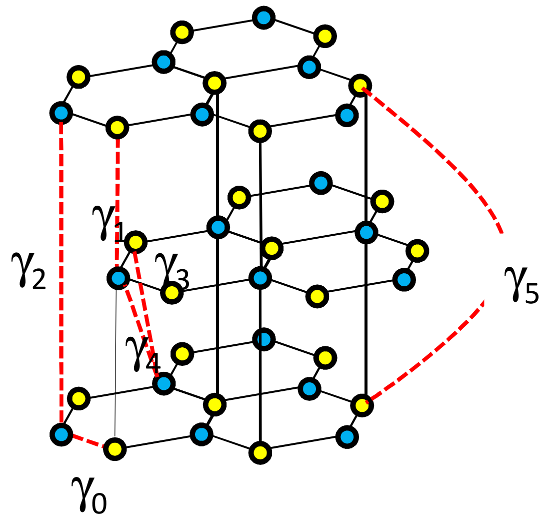

γ1
γ2
γ3
γ4
γ5
γ0

Fig. S9

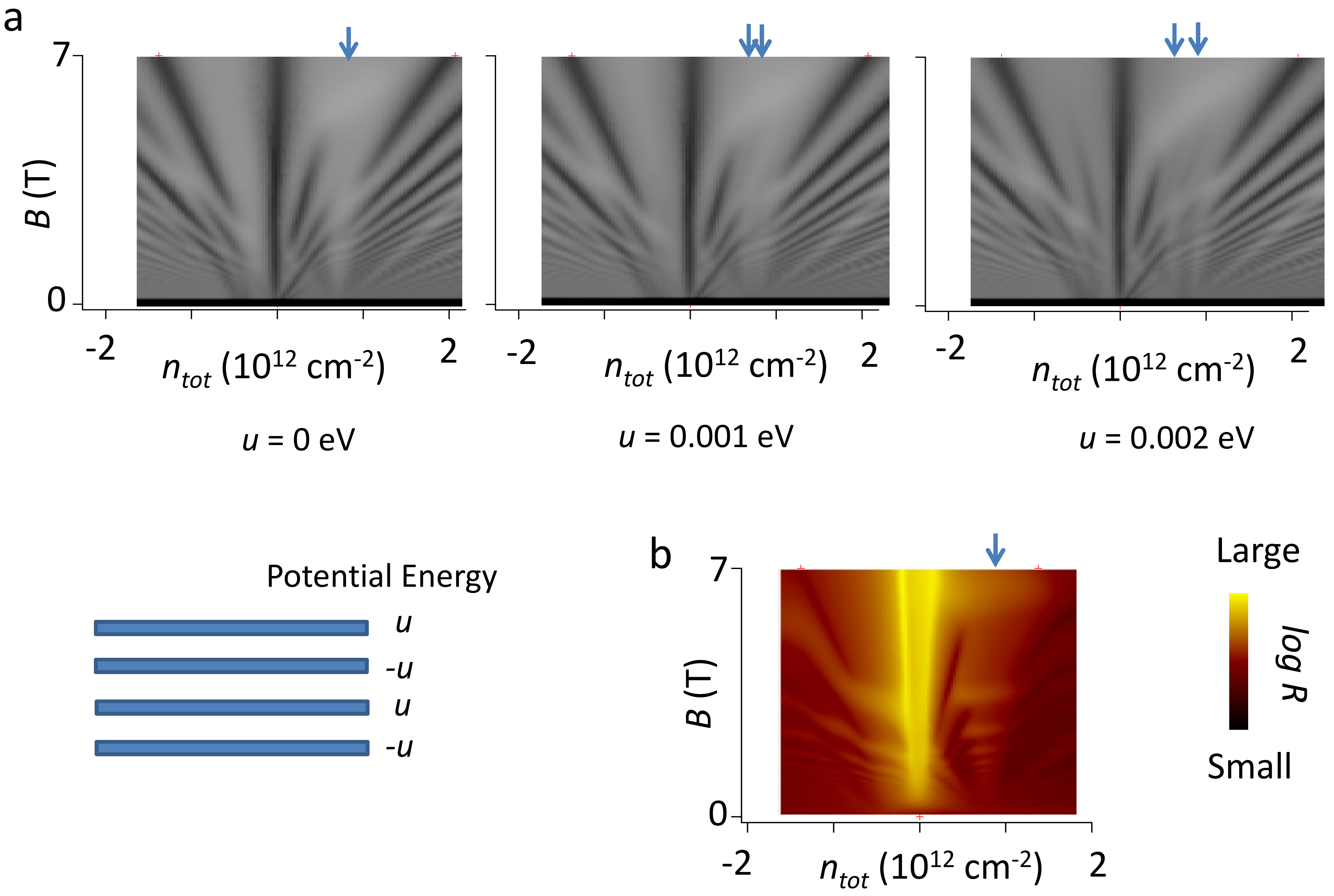
a
7
0
B (T)
-2
2
n_tot (10^12 cm^-2)
u = 0 eV
u = 0.001 eV
u = 0.002 eV
Potential Energy
u
-u
u
-u
b
Large
log R
Small

Fig. S10

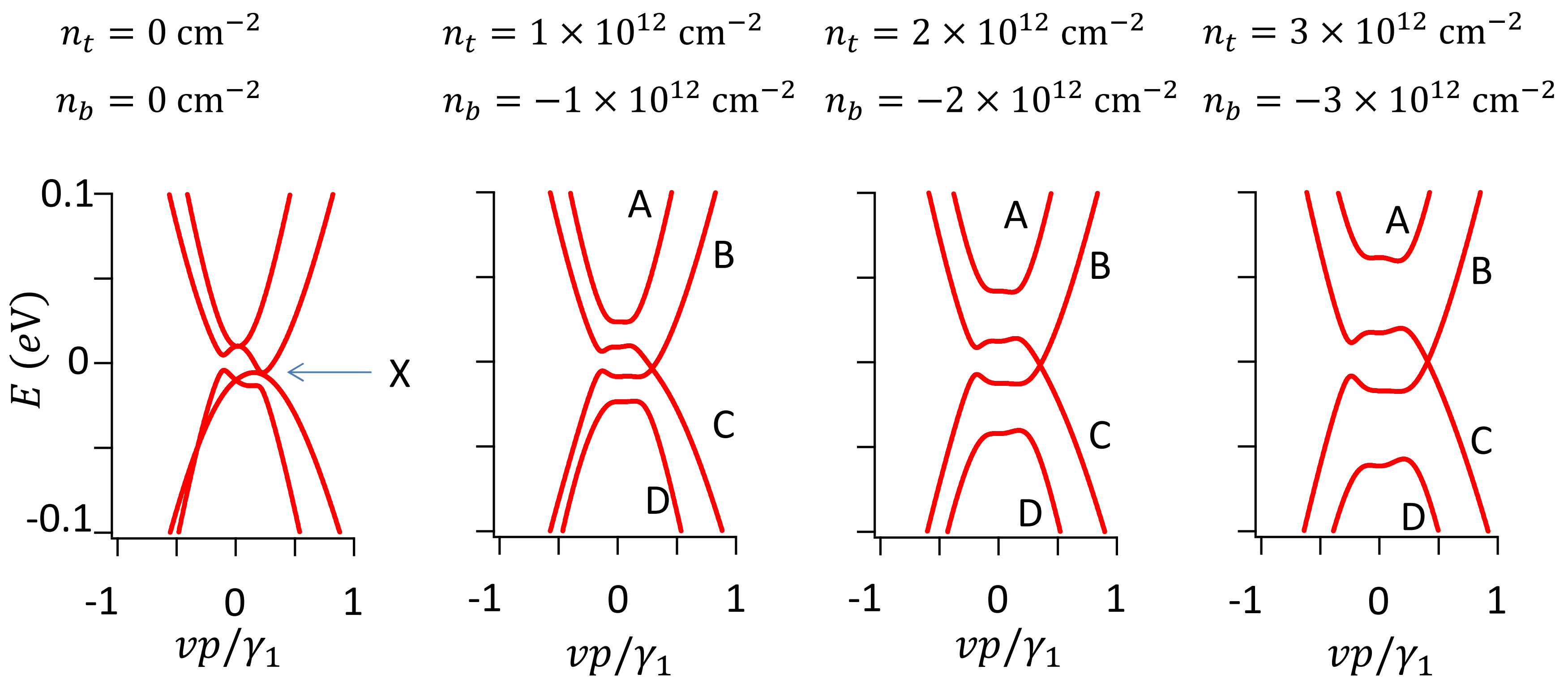

$n_t = 0\ \mathrm{cm}^{-2}$
$n_b = 0\ \mathrm{cm}^{-2}$
$n_t = 1 \times 10^{12}\ \mathrm{cm}^{-2}$
$n_b = -1 \times 10^{12}\ \mathrm{cm}^{-2}$
$n_t = 2 \times 10^{12}\ \mathrm{cm}^{-2}$
$n_b = -2 \times 10^{12}\ \mathrm{cm}^{-2}$
$n_t = 3 \times 10^{12}\ \mathrm{cm}^{-2}$
$n_b = -3 \times 10^{12}\ \mathrm{cm}^{-2}$
0.1
0
-0.1
E (eV)
X
A
B
C
D
-1
0
1
vp/γ_1

Fig. S11

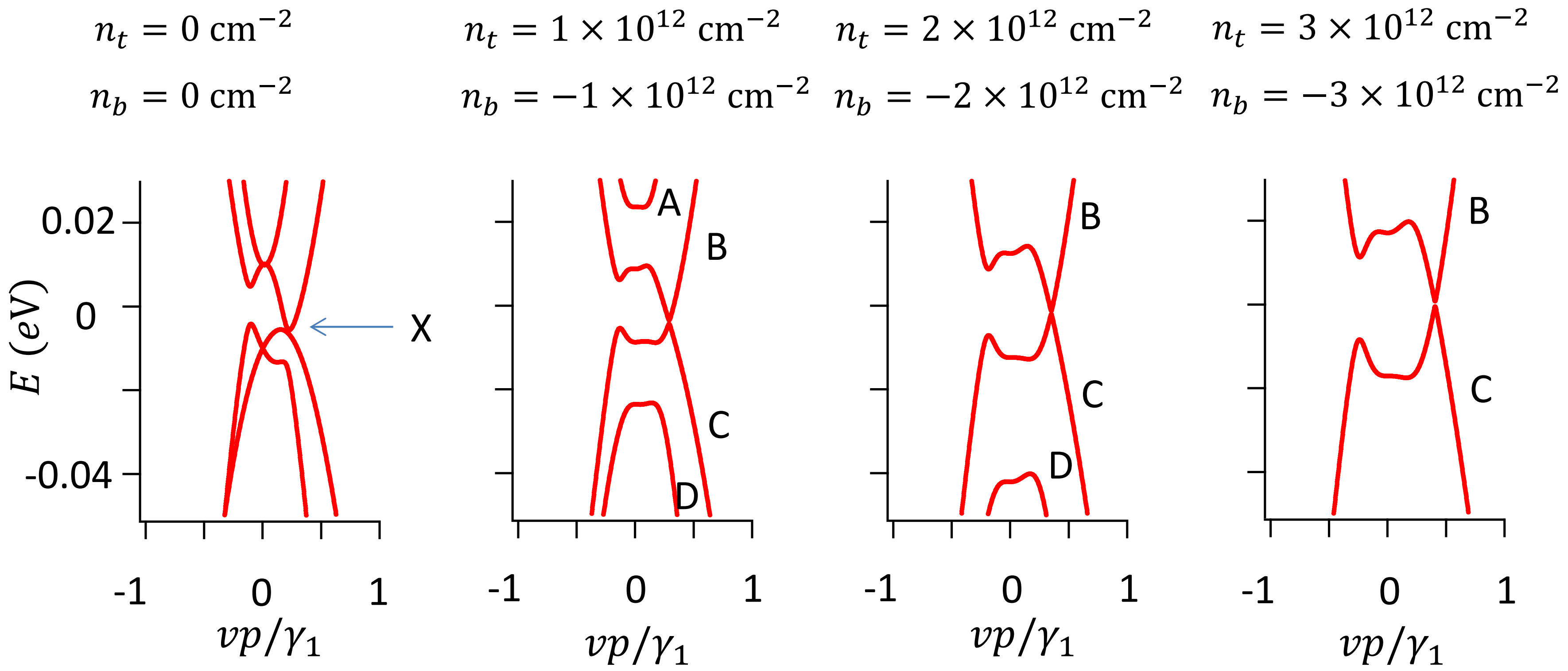

$n_t = 0\ \mathrm{cm}^{-2}$
$n_b = 0\ \mathrm{cm}^{-2}$
$n_t = 1 \times 10^{12}\ \mathrm{cm}^{-2}$
$n_b = -1 \times 10^{12}\ \mathrm{cm}^{-2}$
$n_t = 2 \times 10^{12}\ \mathrm{cm}^{-2}$
$n_b = -2 \times 10^{12}\ \mathrm{cm}^{-2}$
$n_t = 3 \times 10^{12}\ \mathrm{cm}^{-2}$
$n_b = -3 \times 10^{12}\ \mathrm{cm}^{-2}$
$E\ (eV)$
0.02
0
-0.04
X
A
B
C
D
-1
0
1
vp/γ_1

Fig. S12

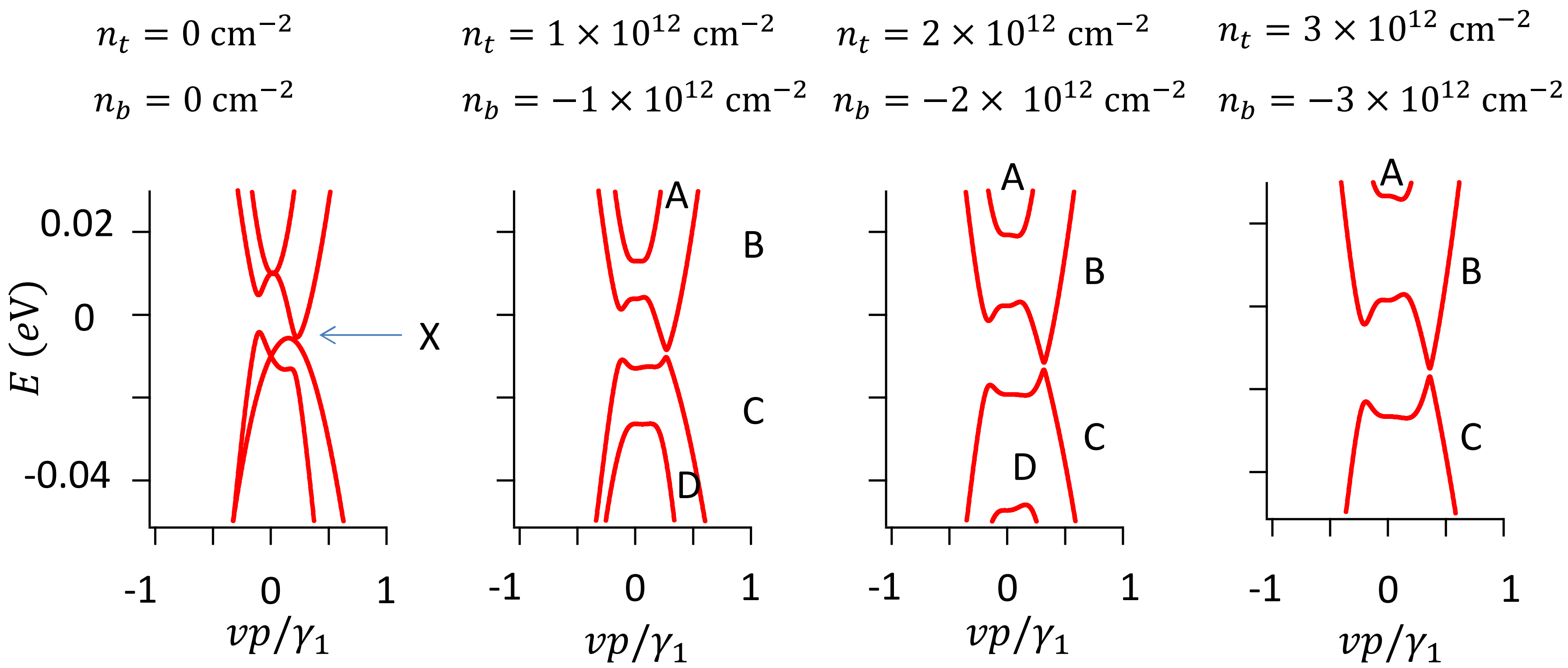

$n_t = 0\ \mathrm{cm}^{-2}$
$n_b = 0\ \mathrm{cm}^{-2}$
$n_t = 1 \times 10^{12}\ \mathrm{cm}^{-2}$
$n_b = -1 \times 10^{12}\ \mathrm{cm}^{-2}$
$n_t = 2 \times 10^{12}\ \mathrm{cm}^{-2}$
$n_b = -2 \times 10^{12}\ \mathrm{cm}^{-2}$
$n_t = 3 \times 10^{12}\ \mathrm{cm}^{-2}$
$n_b = -3 \times 10^{12}\ \mathrm{cm}^{-2}$
$E\ (eV)$
0.02
0
-0.04
X
A
B
C
D
-1
0
1
vp/γ_1

Fig. S13

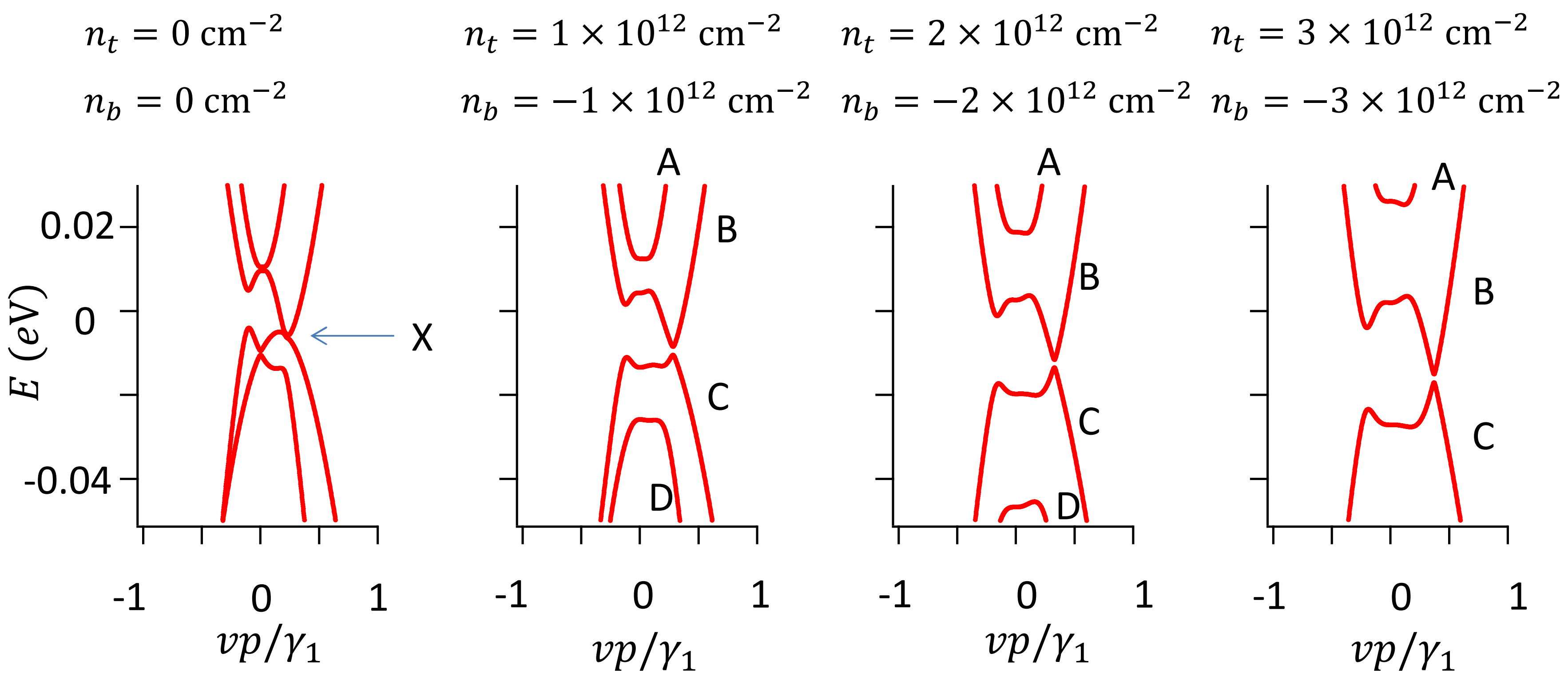
$n_t = 0\ \mathrm{cm}^{-2}$
$n_b = 0\ \mathrm{cm}^{-2}$
$n_t = 1 \times 10^{12}\ \mathrm{cm}^{-2}$
$n_b = -1 \times 10^{12}\ \mathrm{cm}^{-2}$
$n_t = 2 \times 10^{12}\ \mathrm{cm}^{-2}$
$n_b = -2 \times 10^{12}\ \mathrm{cm}^{-2}$
$n_t = 3 \times 10^{12}\ \mathrm{cm}^{-2}$
$n_b = -3 \times 10^{12}\ \mathrm{cm}^{-2}$
E (eV)
0.02
0
-0.04
X
A
B
C
D
-1
0
1
vp/γ_1

Fig. S14

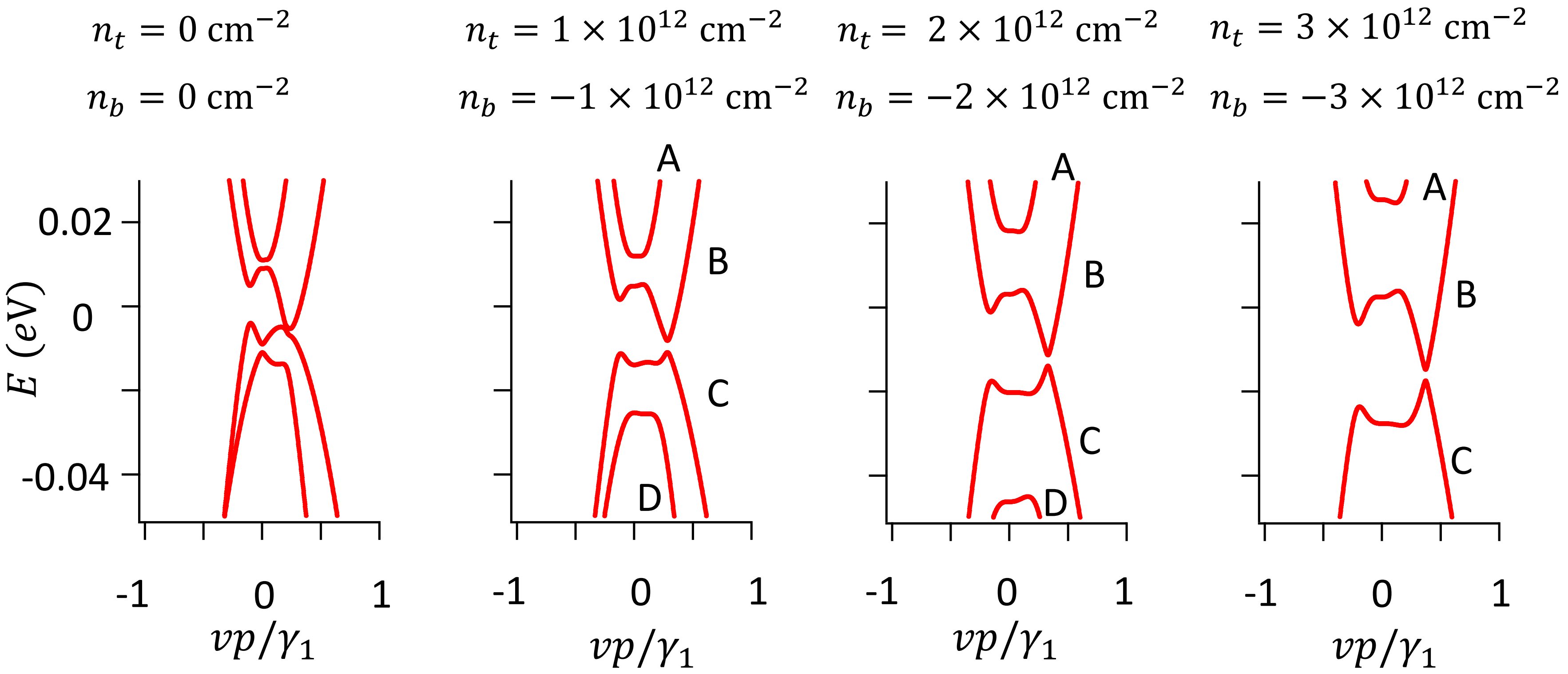

$n_t = 0\ \mathrm{cm}^{-2}$
$n_b = 0\ \mathrm{cm}^{-2}$
$n_t = 1 \times 10^{12}\ \mathrm{cm}^{-2}$
$n_b = -1 \times 10^{12}\ \mathrm{cm}^{-2}$
$n_t = 2 \times 10^{12}\ \mathrm{cm}^{-2}$
$n_b = -2 \times 10^{12}\ \mathrm{cm}^{-2}$
$n_t = 3 \times 10^{12}\ \mathrm{cm}^{-2}$
$n_b = -3 \times 10^{12}\ \mathrm{cm}^{-2}$
$E\ (eV)$
0.02
0
-0.04
A
B
C
D
-1
0
1
vp/γ_1

Fig. S15

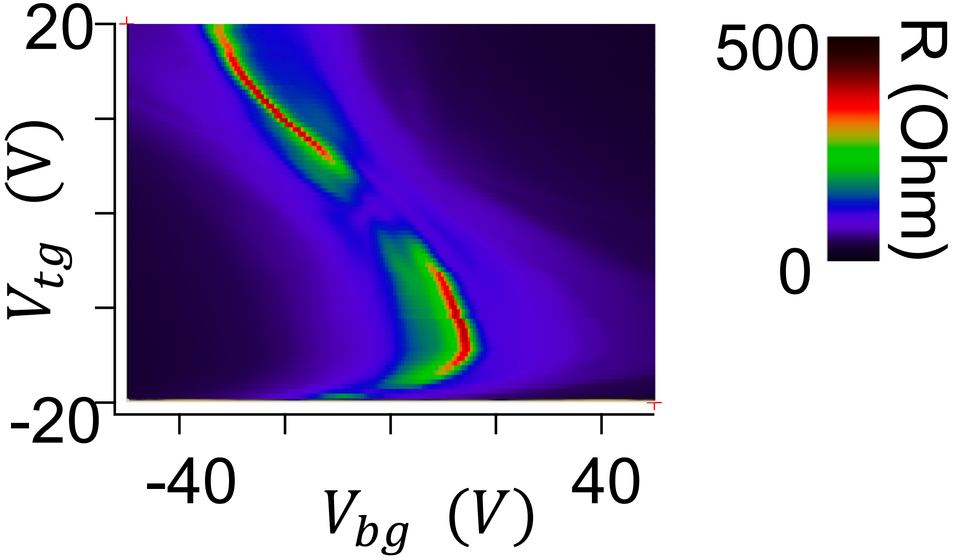

Supplement: Supplementary file 1 — Supplementary Information [file 41598_2018_32214_MOESM1_ESM.pdf]
